# Unraveling the catalytic mechanism of SARS-CoV-2 papain-like protease with allosteric modulation of C270 mutation using multiscale computational approaches†

**DOI:** 10.1039/d3sc00166k

**Published:** 2023-04-11

**Authors:** Qiang Shao, Muya Xiong, Jiameng Li, Hangchen Hu, Haixia Su, Yechun Xu

**Affiliations:** a State Key Laboratory of Drug Research, Shanghai Institute of Materia Medica, Chinese Academy of Sciences Shanghai 201203 China qshao@simm.ac.cn ycxu@simm.ac.cn; b University of Chinese Academy of Sciences Beijing 100049 China; c School of Chinese Materia Medica, Nanjing University of Chinese Medicine Nanjing 210023 China; d School of Pharmaceutical Science and Technology, Hangzhou Institute for Advanced Study, University of Chinese Academy of Sciences Hangzhou 310024 China

## Abstract

Papain-like protease (PL^pro^) is a promising therapeutic target against SARS-CoV-2, but its restricted S1/S2 subsites pose an obstacle in developing active site-directed inhibitors. We have recently identified C270 as a novel covalent allosteric site for SARS-CoV-2 PL^pro^ inhibitors. Here we present a theoretical investigation of the proteolysis reaction catalyzed by the wild-type SARS-CoV-2 PL^pro^ as well as the C270R mutant. Enhanced sampling MD simulations were first performed to explore the influence of C270R mutation on the protease dynamics, and sampled thermodynamically favorable conformations were then submitted to MM/PBSA and QM/MM MD simulations for thorough characterization of the protease-substrate binding and covalent reactions. The disclosed proteolysis mechanism of PL^pro^, as characterized by the occurrence of proton transfer from the catalytic C111 to H272 prior to the substrate binding and with deacylation being the rate-determining step of the whole proteolysis process, is not completely identical to that of the 3C-like protease, another key cysteine protease of coronaviruses. The C270R mutation alters the structural dynamics of the BL2 loop that indirectly impairs the catalytic function of H272 and reduces the binding of the substrate with the protease, ultimately showing an inhibitory effect on PL^pro^. Together, these results provide a comprehensive understanding at the atomic level of the key aspects of SARS-CoV-2 PL^pro^ proteolysis, including the catalytic activity allosterically regulated by C270 modification, which is crucial to the follow-up inhibitor design and development.

## Abstract 2

This theoretical study sheds light on the proteolysis mechanism of SARS-CoV-2 PL^pro^ and how the C270R mutation affects the proteolysis activity, paving the way for understanding the function of PL^pro^ and design of novel allosteric inhibitors.

## Graphical abstract

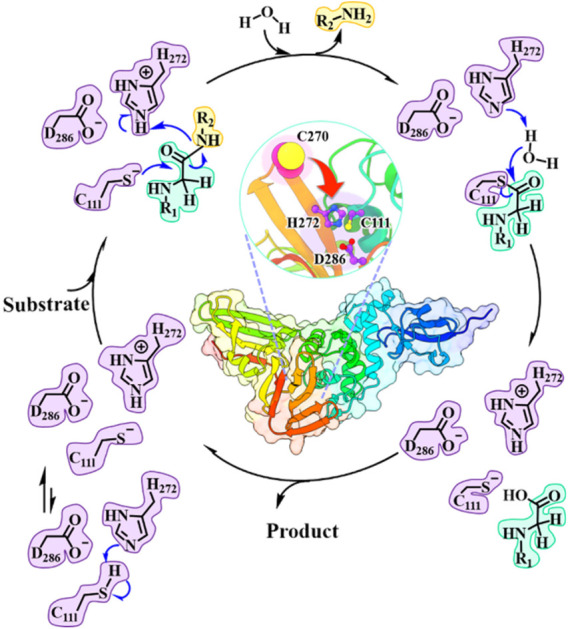

## Introduction

The pandemic of coronavirus disease 2019 (COVID-19) caused by severe acute respiratory syndrome coronavirus 2 (SARS-CoV-2) engenders millions of deaths worldwide, posing extremely huge threats to human society and the global economy. Like SARS-CoV and the Middle East respiratory syndrome coronavirus (MERS-CoV), SARS-CoV-2 utilizes papain-like protease (PL^pro^, a core domain of nsp3) and chymotrypsin-like main protease (3CL^pro^ or M^pro^, nsp5) to process polyproteins to generate non-structural proteins (nsps).^1,2^ While 3CL^pro^ cleaves the polyprotein pp1a/pp1ab at 11 sites with the sequence consensus X-(L/F/M/V)-Q↓(G/A/S/N)-X,^3^ PL^pro^ is responsible for the proteolytic processing of 3 sites with the recognition sequence of LXGG↓XX,^4^ resulting in a total of 16 mature nsps that are essential to the virus proliferation.^5^ Unlike 3CL^pro^ that mainly possesses proteolytic activity, PL^pro^ plays indispensable roles in two other aspects: (1) dysregulating the host immune response, and (2) impairing the antiviral effect of the host type I interferon, owing to its deubiquitinating and deISG15ylating (interferon-stimulated gene 15, ISG15) activities, respectively.^6–8^ PL^pro^ cleaves the ubiquitin or ISG15 modifications aimed at the C-terminal LXGG sequence site to remove them from host cellular proteins, thereby counteracting the host immune response against viral infection.^9^ The LXGG motif corresponds to the P4–P1 amino acids in PL^pro^ substrates. Given the crucial function in not only the processing of viral polyproteins but also disrupting the host antiviral response to facilitate viral proliferation and replication, PL^pro^ has been considered as a promising antiviral target.^4,10–15^

While significant progress has been made in the development of SARS-CoV-2 3CL^pro^ inhibitors and two of them, nirmatrelvir and ensitrelvir, have been approved for the treatment of COVID-19,^16,17^ the development of PL^pro^ inhibitors is currently in early-stage preclinical studies. So far, several types of PL^pro^ inhibitors with moderate inhibitory potency have been identified.^18^ High-throughput screening experiments^4,6,11,19^ found GRL0617 to be one of the most potent SARS-CoV-2 PL^pro^ inhibitors (IC_50_: 2.3 μM)^20^ and the main starting point for further optimization.^10,14^ The phenylthiophene derivatives of GRL0617 improved the inhibitory potency to a nanomolar level.^14^ These small-molecule inhibitors occupied the S3 and S4 subsites of the substrate binding pocket without forming a covalent bond to the catalytic C111. Meanwhile, two covalent peptidomimetic inhibitors, VIR250 and VIR251, fully occupied the S1–S4 subsites of PL^pro^, but showed weak potency with IC_50_ values of ∼50 μM.^4^

PL^pro^ is well conserved in all coronaviruses. For example, SARS-CoV-2 PL^pro^ shares a sequence identity of 83% with SARS-CoV PL^pro^.^21,22^ A collection of recently reported SARS-CoV-2 PL^pro^ structures showed that it adopts a “thumb-palm-fingers” architecture that is constructed by a small *N*-terminal ubiquitin-like (Ubl) domain and a large catalytic domain (Fig. S1 in the ESI†).^6,20^ The latter is an extended right-handed scaffold with three characteristic subdomains of thumb, palm and fingers. A catalytic triad (C111–H272–D286) is located at the interface between the thumb and palm subdomains, neighboring an important BL2 loop (G266–G271) that closes upon substrate or inhibitor binding. Such a characteristic shadow and narrow binding site of PL^pro^ results in featureless S1 and S2 subsites recognizing two consecutive glycines of substrates, thereby posing a great challenge for inhibitor design. In addition, the fact that human deubiquitinases also bind ubiquitin with the consensus sequence LXGG could raise potential concern about the off-target effects of PL^pro^ inhibitors.^18,23^

Recently, we have experimentally observed a novel covalent allosteric site (C270) that can be used to regulate the catalytic activity of SARS-CoV-2 PL^pro^.^24^ The sidechain distance of C270 to the catalytic C111, H272 and D286 is ∼10, ∼9 and ∼14 Å, respectively, in the crystal structure of SARS-CoV-2 PL^pro^ (Fig. S1†). The C270-target covalent binding of the activator or inhibitor did not directly compete for the binding of the substrate to the catalytic site, making C270 act as an allosteric modulation site. In addition, the mutagenesis of C270 substantially influenced the *K*_m_ (Michaelis constant) and *V*_max_ (maximum reaction rate) of SARS-CoV-2 PL^pro^ catalyzing the hydrolysis of the fluorogenic substrate compared to the wild-type (WT) SARS-CoV-2 PL^pro^. *K*_m_ reflects the protease-substrate binding affinity while *V*_max_ is correlated with the reaction rate of the proteolysis. Among 11 C270 mutants, the C270R lowered the *V*_max_ value the most (14.9 ± 2.2 (WT) → 4.7 ± 0.4 (C270R) 10^4^ RFU per min), but raised the value of *K*_m_ (566.9 ± 36 (WT) → 864.5 ± 33.6 (C270R) μM), therefore provoking an inhibitory effect on SARS-CoV-2 PL^pro^. The sequence alignment has revealed that C270 is unique to SARS-CoV-2 and SARS-CoV PL^pro^s and other amino acids such as valine are located at the equivalent position in other PL^pro^s or deubiquitinases.^24^ The design of allosteric inhibitors targeting C270 of PL^pro^ could not only hopefully bypass the difficulty in targeting the narrow orthosteric site but also alleviate the potential problem of off-target effects. Therefore, a thorough understanding of the molecular mechanism underlying such an allosteric inhibition at an atomic level is of interest from both scientific and drug-design viewpoints.

In contrast to extensively reported *in silico* studies of 3CL^pro^ including its catalytic cycle with the substrate and interactions with inhibitors,^25–29^ the computational simulation investigation of PL^pro^ has been limited.^30–32^ In this context, we aimed to shed light on the molecular mechanism of the SARS-CoV-2 PL^pro^ proteolysis by a combination of multiple computational approaches including molecular dynamics (MD) simulations, quantum mechanics/molecular mechanics (QM/MM) MD simulations and molecular mechanics Poisson Boltzmann surface area (MM/PBSA) calculations. The systems investigated include apo and substrate-bound wild-type protease as well as its C270R mutant, with an accumulated simulation time of ∼20 μs for the classical MD simulations and ∼0.2 μs for the QM/MM MD simulations. Moreover, the allosteric modulation induced by the C270R mutation on the catalytic activity of SARS-CoV-2 PL^pro^ was explored through an exhaustive comparative analysis of the structure, dynamics and the free energy profiles associated with the entire proteolysis process. Accordingly, the present study for the first time provides an overview of the reaction energy profile associated with the SARS-CoV-2 PL^pro^ proteolysis and clues about how C270 modification allosterically modulates the proteolytic activity.

## Results and discussion

### Structural plasticity of the active site and effects induced by substrate binding

**Fig. 1:**
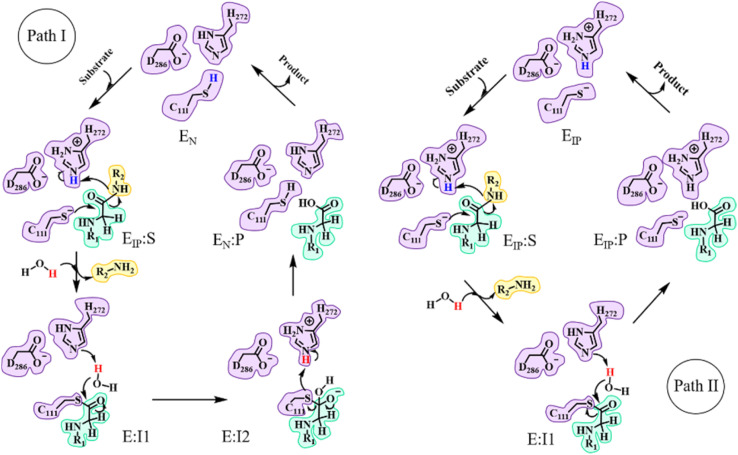
Overall scheme of the proteolysis cycle catalyzed by SARS-CoV-2 PL^pro^. The main divergence is on how the protonation states of C111 and H272 convert during the catalytic reactions, with path I and path II supposing the occurrence of C111–H272 proton transfer after and before the substrate binding, respectively. IP or N in the subscript of each state represents the state containing the ion pair or neutral form of the catalytic C111 and H272.

In the catalytic triad of SARS-CoV-2 PL^pro^, C111 is a nucleophile, H272 acts as a general acid–base, and D286 is paired with H272 so as to promote the deprotonation of C111. Although no experimental evidence has been provided to define the deprotonation process of C111 prior to nucleophilic attack on the substrate, it is generally accepted that the reactive nucleophilic group is the thiolate ion of the thiolate–histidine ion pair (IP) but not the neutral thiol (N).^33–35^ Then a question is raised: does the proton transfer from C111 to H272 occur in the apo enzyme or in the presence of the bound substrate? Accordingly, two paths (I and II) are proposed with the beginning and ending points of the proteolysis cycle containing either the neutral form (C111/H272) or an ion pair (C111^−^/H272H^+^) in the catalytic triad, respectively (Fig. 1). The same issue has been discussed for SARS-CoV-2 3CL^pro^ but the conclusion is under debate.^25,26,36–43^ Though neutron crystallography suggested an ion pair of C145^−^/H41H^+^ adopted in apo SARS-CoV-2 3CL^pro^,^37,38^ an experimental study on the pH-dependent kinetic parameters of SARS-CoV-2 3CL^pro^ and various computational simulations proposed that the apo enzyme favors the neutral form of C145/H41.^25,26,40–43^ One of the simulation studies indicated that the ion pair could perturb the structures of domain I and the active site of SARS-CoV-2 3CL^pro^, and largely impair the pre-reactive binding mode of peptide substrates.^39^ For PL^pro^, so far only one article reported that the deprotonation of C111 for SARS-CoV PL^pro^ occurs during the substrate binding and the proteolysis cycle is completed by recovering the catalytic triad to the neutral form of C111/H272 (as in path I in Fig. 1).^44^

To answer the aforementioned question, we first performed 2-μs Gaussian accelerated MD (GaMD) simulations (with a classical force field) on apo and substrate-bound SARS-CoV-2 PL^pro^ containing the neutral form of C111/H272, respectively. GaMD is a sophisticated enhanced sampling MD method that adds a boosted potential to smoothen the biomolecular potential energy surface that allows for the quantitative measurement of the structural transition of biomolecules with economized computational resources and simulation time.^45–47^ All the MD simulation systems included in the present study are listed in Table 1 and related characterization of these simulation trajectories is shown in Fig. S2–S4.†

**Table 1:** Systems for MD simulations performed using classical force fields and QM/MM MD simulations at the DFTB/MM level^a^

System	PL^pro^	PL^pro^-C270R	PL^pro^-substrate	PL^pro^-C270R-substrate	PL^pro^ (E_IP_)	PL^pro^-C270R (E_IP_)
**GaMD simulations (enzyme structural dynamics evaluation)**						
*N*_atoms_	60 265	60 252	60 289	60 276	60 265	60 252
*t*_simul._ (μs)	2.0	2.0	2.0	2.0	2.0	2.0

**Conventional MD simulations (deacylating water measurement)**						
*N*_atoms_	60 265		61 385	55 255	60 265	
*t*_simul._ (μs)	2.0		2.0	2.0	2.0	

**QM/MM MD simulations**						
*N*_atoms_	60265^a^	60252^a^	60289^a,b^/61385^c,d^	60 276^a,b^/55255^c,d^		
*N*_windows_	13^a^	13^a^	14^a^/24^b^/49^c^/35^d^	14^a^/21^b^/47^c^/33^d^		
*t*_window_ (ps)	500	500	500	500		

a *N*_atoms_: the number of atoms included in each simulation system; *t*_simult._: MD simulation time; *N*_windows_: the umbrella sampling windows used in QM/MM MD simulations for each proteolysis step; *t*_window_: the simulation time per umbrella sampling window. E_IP_ means the PL^pro^ containing the ion pair form of C111 and H272. The substrate is a pentapeptide of Ac-RLRGG-ACC. In QM/MM MD simulations, the detailed numbers of *N*_atoms_ and *N*_windows_ are listed for the systems of C111–H272 proton transfer^a^, acylation^b^, and deacylation following path I^c^ and path II^d^, respectively.

**Fig. 2:**
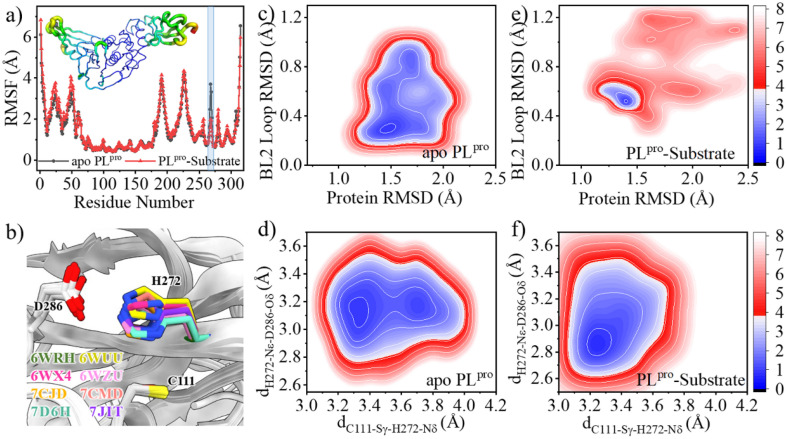
Effects of the substrate binding on the structural dynamics of the BL2 loop and catalytic triad. (a) The comparison of the RMSFs of apo (black) and substrate-bound (red) SARS-CoV-2 PL^pro^. Transparent column marks the position of the BL2 loop. Inset is the RMSF color coded onto the PL^pro^ structure with RGB space (hues from blue to red indicating the increase of RMSF). (b) Superposition of SARS-CoV-2 PL^pro^ crystal structures indicating the rotation of the H272 sidechain with respect to C111. (c–f) Free energy landscapes (FELs) along the main chain root-mean-square deviations (RMSDs) of protein and BL2 loop, the distance between C111-Sγ and H272-Nδ (d_C111-Sγ–H272-Nδ_) and the distance between H272-Nε and D286-Oδ (d_H272-Nε–D286-Oδ_) in GaMD simulations of (c, d) apo and (e, f) substrate-bound PL^pro^, respectively. The RMSD is according to the apo crystal structure (PDB code 6WRH). The contours in the two-dimensional subspace are spaced at intervals of 1.0 kcal mol^−1^.

The root-mean-square fluctuations (RMSFs) per residue show that in the entire PL^pro^ structure, the Ubl and fingers subdomains as well as the BL2 loop in the palm subdomain display high dynamic features (Fig. 2a). Previous studies showed that the BL2 loop at the entrance of the active site shows a high plasticity in SARS-CoV PL^pro^, with a more open conformation responsible for the binding of a larger inhibitor.^44^ Here, it is also showed that the substrate binding affects the BL2 loop of SARS-CoV-2 PL^pro^. As shown in the free energy landscape (FEL) produced from the GaMD simulation (Fig. 2c), while the BL2 loop in the crystal structure of apo PL^pro^ is most energetically favored (1.3 Å < protein RMSD < 1.6 Å and 0.2 Å < BL2 loop RMSD < 0.4 Å), it is also capable of visiting a large conformational space. The substrate binding, however, confines its structure into a local minimum slightly different from the apo structure (1.2 Å < protein RMSD < 1.6 Å and 0.5 Å < BL2 loop RMSD < 0.7 Å, Fig. 2e).

The substrate binding affects not only the BL2 loop but also the catalytic triad. The neutral H272 is supposed to be protonated at the Nε position in order to form a hydrogen bond with the sidechain carboxy group of D286, thus stabilizing the configuration of the catalytic triad, while its Nδ atom is ready to accept the proton transferred from C111. The distance between C111-Sγ and H272-Nδ atoms (d_C111-Sγ–H272-Nδ_) and the distance between H272-Nε and D286-Oδ (d_H272-Nε–D286-Oδ_) were thus used as collective variables (CVs) to evaluate the structural dynamics of the catalytic triad. In the FEL of apo PL^pro^, two local minima (3.2 Å < d_C111-Sγ–H272-Nδ_ < 3.4 Å and 3.0 Å < d_H272-Nε–D286-Oδ_ < 3.3 Å, 3.6 Å < d_C111-Sγ–H272-Nδ_ < 3.8 Å and 3.0 Å < d_H272-Nε–D286-Oδ_ < 3.3 Å) are presented (Fig. 2d). This suggests that the sidechain distance between the two neutral residues of C111 and H272 is rather flexible, due to the flexible orientation of the H272 imidazole ring. Intriguingly, the fact that the H272 sidechain rotates to alter its distance to C111-Sγ has been shown in multiple crystal structures of SARS-CoV-2 PL^pro^ (Fig. 2b). In contrast, the binding of substrate confines the catalytic triad to a tightly contacted configuration (3.2 Å < d_C111-Sγ–H272-Nδ_ < 3.3 Å and 2.8 Å < d_H272-Nε–D286-Oδ_ < 3.0 Å, Fig. 2f).

### C111–H272 proton transfer

A pioneering QM/MM study using the B3LYP functional and TZVP basis set suggested that the substrate binding remarkably facilitates the C145–H41 proton transfer in SARS-CoV 3CL^pro^ by causing a reduction of ∼7.0 kcal mol^−1^ in the free energy level for the ion pair (C145^−^/H41H^+^) relative to the neutral form (C145/H41).^48^ In contrast, in the QM/MM MD simulation of SARS-CoV-2 3CL^pro^ with the B3LYP functional and 6-31+G* basis set, the substrate binding makes the C145–H41 proton transfer more difficult by increasing the free energy level of the ion pair by ∼1.9 kcal mol^−1^.^26^ These computational studies, although being different from each other in the results, indicate that the substrate binding would affect the cysteine–histidine proton transfer in cysteine proteases.

In the present study, the most populated structure of either apo or substrate-bound SARS-CoV-2 PL^pro^ for QM/MM MD calculations was extracted from the microsecond-timescale GaMD simulations. The QM/MM free energy profiles associated with the proton transfer and other reactions hereinafter were explored at the DFTB/MM level. The use of a similar tight-binding DFT method (DFTB3) to describe the QM region resulted in a geometrical description of the SARS-CoV-2 3CL^pro^–inhibitor reaction in good agreement with the DFT (*e.g.*, B3LYP and M06-2X) results and reasonable evaluation of the activation free energies through single-point energy correlations using higher-level QM methods.^49^ In the QM/MM MD simulations, steered MD (SMD) simulations were run along specific reaction coordinates to collect the structures along the reaction path, and umbrella sampling (US) simulations were performed on the chosen structures to enhance the sampling of the reaction configuration space and explore the free energy profiles.

Before the detailed analysis, we first evaluated the factors affecting the QM/MM MD simulations, which are, namely, the starting structure and the cutoff value used for treating nonbonding interactions. We tested 3 different starting structures randomly chosen from the populated structure cluster identified in the GaMD simulation, and the yielded QM/MM free energy profiles of the C111–H272 proton transfer and acylation reaction are converged with each other, respectively (Fig. S5a and b†). Additionally, the often used QM/MM cutoff value is in the range of 8 ∼ 15 Å.^25,26,50,51^ We tried different cutoff values (8, 12 and 14 Å) to evaluate the free energy barrier of the E-I1 → E-I2 sub-step of the deacylation reaction, and the resulting free energy profiles are very close to each other (Fig. S5c†). Therefore, the two abovementioned factors might have trivial influence on the QM/MM MD simulations under study.

**Fig. 3:**
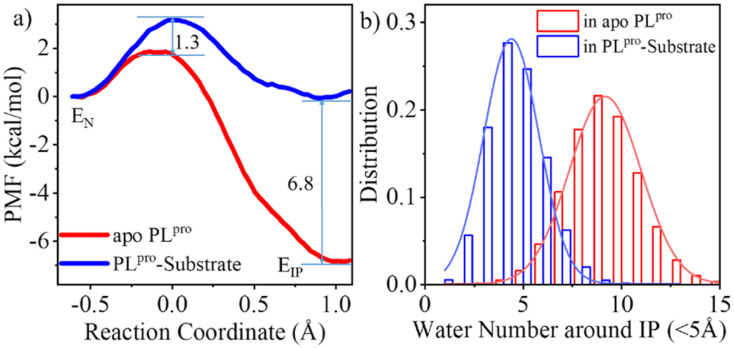
Effects of the substrate binding on the C111–H272 proton transfer. (a) The QM/MM MD calculations of free energy profiles associated with the C111–H272 proton transfer for apo (red) and substrate-bound PL^pro^ (blue), in terms of potentials of mean force (PMFs). E_N_ or E_IP_ means the PL^pro^ containing the neutral or ion pair form of C111 and H272, respectively. The reaction coordinate of PMF is defined by the distance difference of Sγ-H to Nδ-H (d_C111-Sγ-H_ − d_H272-Nδ-H_). (b) Number of water molecules around the C111^−^/H272H^+^ ion pair in apo and substrate-bound SARS-CoV-2 PL^pro^.

As shown in Fig. 3a, the QM/MM calculated free energy barrier for the proton transfer from C111 to H272 and the free energy level of the ion pair in apo PL^pro^ are 1.3 and 6.8 kcal mol^−1^ lower than those in the substrate-bound PL^pro^, respectively. These results strongly suggest that apo SARS-CoV-2 PL^pro^ predominantly contains the C111^−^/H272H^+^ ion pair. Accordingly, the GaMD simulation on apo PL^pro^ containing the ion pair obtained a steady compact catalytic triad configuration (2.8 Å < d_C111-Sγ–H272-Nδ_ < 3.2 Å and 3.1 Å < d_H272-Nε–D286-Oδ_ < 3.6 Å) and a rigid BL2 loop (BL2 loop RMSD < 0.4 Å) (Fig. S6a†). More water molecules were found around the C111^−^/H272H^+^ ion pair in apo PL^pro^ compared to the substrate-bound PL^pro^ (Fig. 3b). The worse solvation of the catalytic triad in the presence of the bound substrate, as suggested by the previous QM/MM MD simulations of SARS-CoV-2 3CL^pro^,^26^ explains the substrate-binding induced increase in the free energy level of the ion pair form.

The free energy barrier associated with the proton transfer of SARS-CoV-2 PL^pro^ (1.9 kcal mol^−1^ in apo or 3.2 kcal mol^−1^ in substrate-bound PL^pro^) is smaller than the previously reported values for SARS-CoV-2 3CL^pro^ (*e.g.*, ∼5.6 or 6.1 kcal mol^−1^ in apo or substrate-bound 3CL^pro^ in ref. 26). Precluding the influence from the usage of different methodologies, the lower free energy barrier makes the proton transfer easier to occur in SARS-CoV-2 PL^pro^ compared to SARS-CoV-2 3CL^pro^, owing to the assistance of D286 in the catalytic triad of PL^pro^. It is interesting that water molecules have been detected in the active site of SARS-CoV-2 3CL^pro^. A recent study using the ProBiS H_2_O approach to investigate water molecules within 72 3CL^pro^ crystal structures observed four conserved water molecules,^52^ one of which is near H41 and may mediate formation of hydrogen bonds with H41 and D187.^16^ To evaluate the interactions of H41 with this water molecule, we ran conventional MD simulations on apo SARS-CoV-2 3CL^pro^ containing either the neutral or ion pair form of the catalytic dyad. The resulting FEL (Fig. S7†) shows that the water molecule favors the location between H41 and D187, with short distances from its oxygen to both H41-Nδ and D187-Oδ atoms, but it also moves away from either or both residues, similar to the observed results in previous MD simulations on MERS-CoV and SARS-CoV 3CL^pro^.^36^ Therefore, even though the water molecule might help with the general acid–base function of H41 in catalysis by forming water-mediated hydrogen bonds, the stability deficiency limits its influence on H41 in SARS-CoV-2 3CL^pro^, as compared to the sustained contribution of D286 to H272 in SARS-CoV-2 PL^pro^.

### Acylation and deacylation steps

**Fig. 4:**
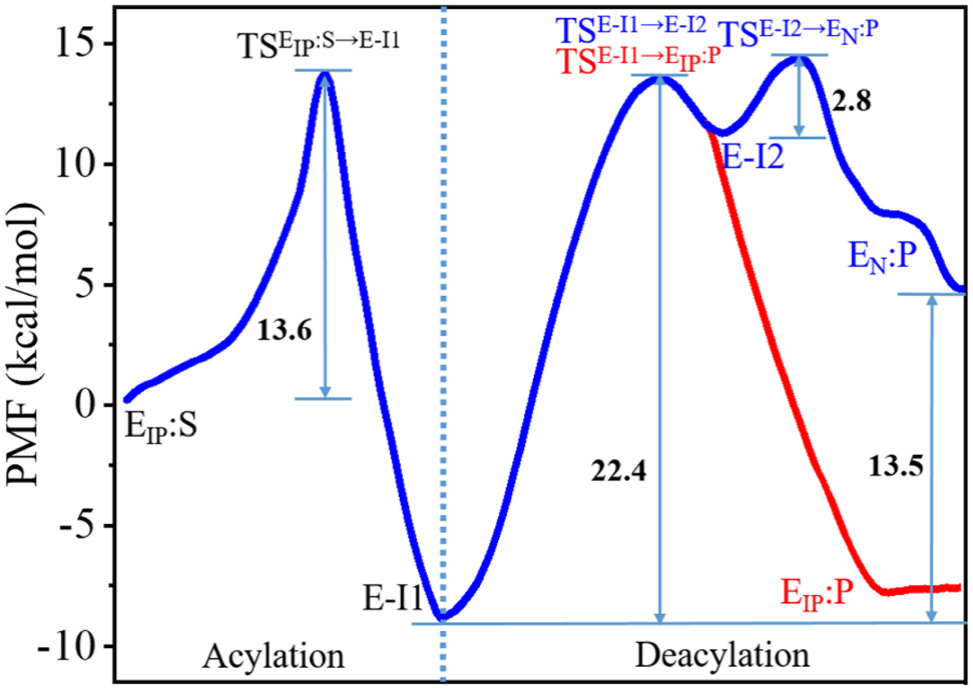
Free energy profiles associated with the acylation and deacylation reactions for the SARS-CoV-2 PL^pro^ proteolyzing substrate resulting from the QM/MM MD simulations. The profiles were calculated following the proposed paths I and II in Fig. 1, colored by blue and red, respectively. The profiles are identical for the acylation and a part of deacylation in the two paths. Important states are presented: the PL^pro^-substrate complex after the C111–H272 proton transfer (E_IP_:S), the transition state (TS^E_IP_:S→E-I1^) and intermediate (E-I1) in the acylation step that are in common in paths I and II (black letters); the transition states (TS^E-I1→E-I2^ and TS^E-I2→EN:P^), intermediate (E-I2), and product (E_N_:P) in the deacylation step of path I (blue letters); the transition state (TS^E-I1→E_IP_:P^) and product (E_IP_:P) in the deacylation step of path II (red letters).

The C111-Sγ atom maintains tight contact with the P1-C atom and the Nδ-H proton of H272H^+^ is close to the P1′-N atom in the GaMD simulation of SARS-CoV-2 PL^pro^ in complex with the substrate (Fig. S8†), ready for the nucleophilic attack of Sγ to P1-C and the proton transfer from H272H^+^ towards P1′-N to generate the P′-NH_2_ fragment. The QM/MM calculated free energy profiles for the acylation and deacylation reactions involved in the proteolysis cycle of SARS-CoV-2 PL^pro^ are shown in Fig. 4. The acylation occurring between PL^pro^ and the substrate maintains a single transition state (TS^E_IP_:S→E-I1^). Two transition states (TS^E-I1→E-I2^ and TS^E-I2→E_N_:P^) exist in the deacylation reaction in the case that the final product E_N_:P contains the neutral form of C111/H272 (path I in Fig. 1), whereas the second transition state vanishes if the ion-paired E_IP_:P is produced (path II). It should be noted that path I seems to be highly endergonic with the free energy of E_N_:P being 13.5 kcal mol^−1^ above that of E-I1, giving rise to a non-favored thermodynamic process in which the enzyme could be inhibited by an accumulation of the acyl-enzyme. In contrast, the free energy difference is significantly decreased to 1.2 kcal mol^−1^ in path II. The small free energy difference of the product relative to the reactant in the deacylation can also be seen in a number of QM/MM MD simulations on other cysteine proteases (*e.g.*, 5.2 kcal mol^−1^ for human cathepsin K,^53^ −0.6 kcal mol^−1^ for papain,^54^ and −0.8 kcal mol^−1^ for cruzain cysteine protease^35^). In this scenario, the catalytic triad would stay in the ion pair form (C111^−^/H272H^+^) at the end of the deacylation and keep it for the next proteolysis cycle. The mechanistic investigation will thus focus on path II hereinafter.

While proton transfer is a straightforward process, the acylation and deacylation steps are complex. The reaction coordinates (RC) used for PMF calculation are: RC = (d_C111-Sγ–P1-C_ + d_H272-Hδ–P1′-N_) for the transition of E_IP_:S → E-I1 (Fig. S9†); RC = (d_H272-Nδ–water-H_ + d_water-O–P1-C_ − d_water-O–water-H_) for E-I1 → E-I2 (Fig. S10†); RC = (d_C111-Sγ–P1-C_ − d_C111-Sγ–H272-Hδ_ + d_H272-Hδ–H272-Nδ_) for E-I2 → E_N_:P (Fig. S11†); RC = (d_H272-Nδ–water-H_ + d_water-O–P1-C_ − d_water-O–water-H_ − d_C111-Sγ–P1-C_) for E-I1 → E_IP_:P. It should be noted that a good reaction coordinate could avoid hysteresis problems.^55–57^ Using the transition of E-I1 → E-I2 as an example, we tested multiple reaction coordinates to evaluate the hysteresis effects. It can be seen from Fig. S12† that, no matter what reaction coordinates are used, the non-equilibrium work drawing the reactants to products in the SMD simulations is almost identical. These SMD work profiles have features similar to the corresponding E-I1 → E-I2 PMF profile (Fig. S5c†). Moreover, individual distances selected as CVs change with the similar tendencies in the three SMD yielded paths, suggesting that the reaction paths are also identical (Fig. S12†). Thus, the energetic properties of the reaction do not appear to depend on the reaction coordinates used or suffer hysteresis. On the other hand, Fig. S9–S11† show that in each of the three abovementioned transitions, the evolution of individual CVs is asynchronous, implying that there is less probability of the present simulations yielding artificial concerted steps for complex reactions.

The free energy level of the intermediate E-I1 (acyl-enzyme complex) is much lower than that of the E_IP_:S Michaelis complex and accordingly, the free energy barrier in the deacylation step is significantly higher than that in the acylation step, suggesting deacylation is the rate-limiting step in the entire proteolysis process. Such a feature of the free energy profile of SARS-CoV-2 PL^pro^ is similar to that of SARS-CoV-2 3CL^pro^ resulting from various calculation methods.^25–27^

**Fig. 5:**
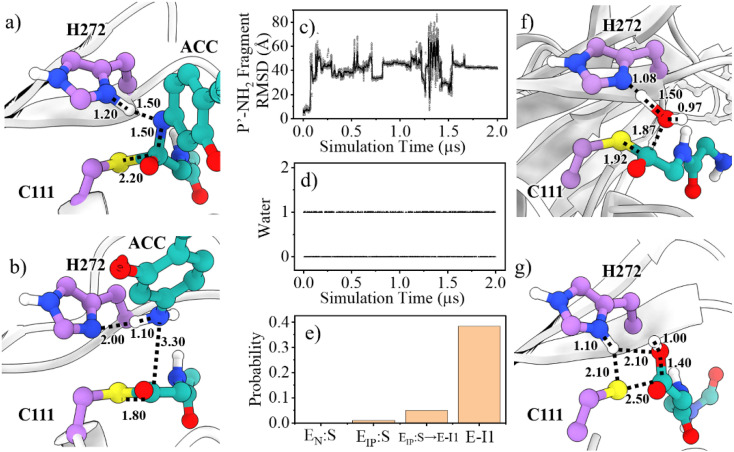
Molecular mechanism of the acylation and deacylation reactions of SARS-CoV-2 PL^pro^. (a and b) Representative structures of TS^E_IP_:S→E-I1^ and E-I1 states in the acylation reaction. (c) Representative MD trajectory indicating the movement of the P′-NH_2_ fragment away from the enzyme. (d) The MD trajectory indicating the existence of a deacylating water molecule. (e) The existing probability of the deacylating water in various stages (E_IP_:S → E-I1 represents the acylation process measured in QM/MM MD simulations). (f and g) Structures of TS^E-I1→E_IP_:P^ and E_IP_:P in the deacylation step, respectively. Distances are labelled in angstrom.

Collection of the QM/MM umbrella sampling trajectories shows that in the acylation reaction, the nucleophilic attack of P1-C by C111-Sγ and the associated breakage of the P1-C–P1′-N bond in the substrate follow the proton transfer from H272H^+^ to the P1′-N atom. As a result, in the TS^E_IP_:S→E-I1^ state, the Nδ-H proton in H272H^+^ approaches the substrate P1′-N atom, slightly increasing the Nδ–H bond distance (1.09 → 1.20 Å, Fig. 5a). Meanwhile, the C111-Sγ atom also stays closer to the P1-C atom (2.56 → 2.20 Å) and the peptide bond P1-C–P1′-N distance is increased from 1.41 to 1.50 Å. This process is roughly identical to the acylation step of SARS-CoV-2 3CL^pro^ described in a previous QM/MM MD simulation.^26^ The free energy barrier associated with the acylation of SARS-CoV-2 PL^pro^ (13.6 kcal mol^−1^) is also close to that of SARS-CoV-2 3CL^pro^ (14.6 kcal mol^−1^) calculated by using the combination of the B3LYP functional and 6-31+G* basis set^26^ but relatively lower than that of SARS-CoV-2 3CL^pro^ (19.9 kcal mol^−1^) calculated by using the M06-2X functional and semi-empirical AM1 method.^25^ The higher free energy barrier in the latter literature is attributed to the suggested different mechanism in which the acylation step begins with the neutral form of the catalytic dyad and the nucleophilic attack of the substrate P1-C atom by C145-Sγ is concomitant with the proton transfer from C145 to H41.^25^

In addition to the separated C111–H272 proton transfer and the nucleophilic attack of P1-C by C111-Sγ, other possible mechanisms of acylation of SARS-CoV-2 PL^pro^ were also explored. First, the case whether the attack of P1-C by C111-Sγ could be concerted to the C111–H272 proton transfer as the acylation starts from an E_N_:S complex carrying a neutral form of C111/H272 was examined. In this way, a transient thiohemiketal (THA) state would be formed, which then would undergo P1-C–P1′-N bond breakage assisted by proton transfer from H272H^+^ to P1′-N, forming an E-I1 intermediate and releasing a P1′-NH_2_ fragment (Fig. S13a†). The calculated free energy of the THA state is significantly higher than that of E_N_:S (22.6 kcal mol^−1^) and the free energy barrier for this stepwise acylation is also large (30.5 kcal mol^−1^) (Fig. S13b†). The analysis of the reaction path shows that the C111–H272 proton transfer occurs distinctly prior to the attack of P1-C by C111-Sγ (Fig. S13b and c†). Second, an acylation with a C111-Sγ attacking the substrate P1-C concerted to the proton transfer from C111-Hγ to P1′-N was also investigated. The free energy barrier for such a concerted process is also significantly higher than that in the acylation step starting with the ion pair of C111^−^/H272H^+^ (20.6 *vs.* 13.6 kcal mol^−1^) (Fig. S13e–g†*vs.*Fig. 4). Similar results have been observed in the QM/MM MD simulations of SARS-CoV-2 3CL^pro^.^26^ These results together suggest that a separated C111–H272 proton transfer and nucleophilic attack of P1-C by C111-Sγ occurs in the PL^pro^ proteolysis.

A P′-NH_2_ fragment (ACC-NH_2_) is yielded in the acylation step (Fig. 5b). Subsequently, a water molecule at the position neighboring H272 and the substrate P1-C atom is supposed to be involved in the deacylation reaction. This water molecule is activated by hydrogen bonding to H272 and then its oxygen atom attacks the P1-C atom, releasing the P-COOH fragment, and regenerating the free catalytic triad (path II in Fig. 1). Unlike SARS-CoV-2 3CL^pro^ that contains a highly conserved water molecule at a position compatible for serving as the deacylating nucleophile in the high-resolution crystal structure of the acyl-enzyme intermediate,^58^ such a water molecule has not been found in crystal structures of SARS-CoV-2 PL^pro^ yet.

To inspect whether the P′-NH_2_ fragment stays steadily around the active site of PL^pro^ and the presence of the potential deacylating water molecule, we ran 2-μs conventional MD (cMD) simulations on the acyl-enzyme (E-I1 state) yielded in the acylation step. It can be seen from the cMD trajectory (Fig. 5c) that the P′-NH_2_ fragment moves away from PL^pro^, thus it is not included in the subsequent QM/MM MD calculations. On the other hand, a deacylating water molecule continuously exists in between H272 and the P1-C, with an existing probability of 38.4% or 52.9% in the last half stage of the 2-μs simulation trajectory of the wild-type or C270R mutant of the acyl-enzyme complex (Fig. 5d and S14†), respectively. The existing probability of such a water molecule was also calculated in various other stages, which is close to 0 in the Michaelis complex and slightly increased during the acylation reaction and reaches the maximum in the acyl-enzyme complex (Fig. 5e). This implies the existence of the deacylating water molecule that might come inside the binding pocket when the P′-NH_2_ fragment product is released after the acylation step.

The deacylation yielding ion-paired catalytic triad proceeds *via* a single transition state (Fig. 4). In the representative structure of the transition state, the water oxygen atom approaches the P1-C atom (1.87 Å) but the bond is not formed yet while the proton transfer from the water molecule to H272 is completed (Fig. 5f). Finally, at the E_IP_:P state, the hydroxyl group and the carbonyl carbon is bound with a distance of 1.40 Å and the C111-Sγ–P1-C bond is broken (2.50 Å), regenerating the protease with a C111^−^/H272H^+^ ion-paired catalytic triad and yielding the P-COOH fragment (Fig. 5g). This transition step has to overcome a free energy barrier of 22.4 kcal mol^−1^, a value higher than the counterpart calculated in the B3LYP/6-31+G* simulation (15.6 kcal mol^−1^)^26^ but very close to that in the M06-2X/AM1 simulation for SARS-CoV-2 3CL^pro^ (22.8 kcal mol^−1^).^25^ The latter literature suggested a similar deacylation mechanism in which the water hydrogen is attracted by H41 of 3CL^pro^ to let the water oxygen attack the P1-C atom,^25^ whereas the former literature proposed that the water hydrogen is attracted by the P′-NH_2_ fragment released in the acylation step.^26^

### C270R mutation changing the structural dynamics of the active site

As mentioned above, the SARS-CoV-2 PL^pro^ proteolysis includes proton transfer from C111 to H272 with the help of D286 before the substrate binding, and when bound with the substrate, the acylation step is followed by the deacylation step that releases the products and regenerates the free catalytic triad containing the C111^−^/H272H^+^ ion pair. Among these steps, the deacylation step is the rate-limiting one as its free energy barrier is the largest. With this elucidated mechanism of the proteolysis, it is possible to investigate the molecular mechanism underlying C270R mutation interfering with the catalytic activity of the protease. As the crystal structure of the C270R mutated SARS-CoV-2 PL^pro^ is not available, we ran GaMD simulations on this mutated protease in an apo and a substrate-bound state, respectively, to obtain the equilibrated structures and investigate the structural dynamic features under the mutation.

**Fig. 6:**
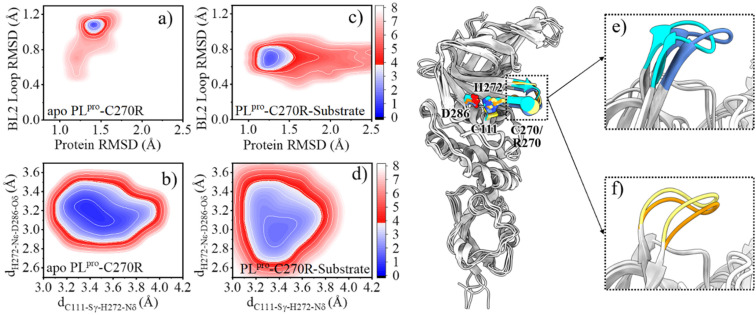
The comparison of the structure and dynamics of the wild-type (WT) and C270R mutant of PL^pro^. (a–d) FELs along the protein and BL2 loop RMSDs, the distance between C111-Sγ and H272-Nδ (d_C111-Sγ–H272-Nδ_) and the distance between H272-Nε and D286-Oδ (d_H272-Nε–D286-Oδ_) for GaMD simulations of (a, b) the apo and (c, d) substrate-bound complex of PL^pro^-C270R mutant (PL^pro^-C270R), respectively. The contours in the two-dimensional subspace are spaced at intervals of 1.0 kcal mol^−1^. (e and f) The comparison of the BL2 loop structures in apo wild-type PL^pro^ (blue) and PL^pro^-C270R (cyan), and substrate-bound wild-type PL^pro^ (yellow) and PL^pro^-C270R (orange). The catalytic triad and C270/R270 residues are shown as sticks and spheres, respectively.

The comparison of the FELs (Fig. 2c–f and 6a–d) suggests that the C270R mutation induces the conformational change of the BL2 loop, generating a more hooked structure in the apo protease (see the local minimum of 1.3 Å < protein RMSD < 1.6 Å and 1.0 Å < BL2 loop RMSD < 1.2 Å in Fig. 6a and loop structure in Fig. 6e). The binding of the substrate, on the other hand, drives the BL2 loop to become more open to adopt the substrate peptide (Fig. 6c). And a trivial difference still exists between the BL2 loop structures of the substrate-bound wild-type and C270R mutated PL^pro^ (Fig. 6f). Therefore, the structure and dynamics of the BL2 loop are influenced by the C270R mutation.

### C270R mutation reducing the binding affinity of PL^pro^ with the substrate

**Fig. 7:**
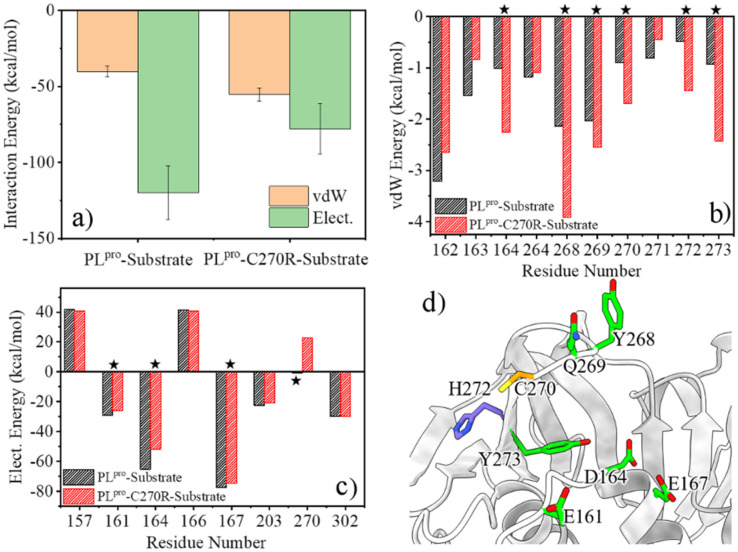
The calculated protease-substrate interaction energy difference is consistent with the *K*_m_ difference between the wild-type (WT) and C270R mutant of SARS-CoV-2 PL^pro^. (a) The comparison of the protease-substrate vdW and electrostatic interaction energies between the WT and C270R mutant. (b and c) Decomposition of the vdW and electrostatic interaction energies into individual residues. Only residues with strong vdW or electrostatic energy are presented (*e.g.*, per-residue vdW energy < −1.0 kcal mol; electrostatic energy < −20 kcal mol^−1^ or >20 kcal mol^−1^). Residues displaying obvious energy changes are marked by stars. (d) The location of the residues which impact the protease-substrate interaction energy difference between the WT and C270R mutant of SARS-CoV-2 PL^pro^.

Any change in the structure and/or dynamics of the active site could directly influence the substrate binding. The most populated PL^pro^-substrate complex structure was clustered from the GaMD trajectories for the wild-type or C270R mutant and used for MM/PBSA calculations to evaluate the binding interactions. It can be seen from Fig. 7a that the protease-substrate vdW interaction energy is increased whereas the electrostatic interaction energy is obviously decreased for the C270R mutant compared with the wild-type protease. As a result, the binding interaction of the mutant with the substrate is weakened as compared to the wild-type, consistent with the increased *K*_m_ value of the mutant (864.5 *vs.* 566.9 μM for C270R *vs.* WT).^24^ The energy decomposition indicates that the increased vdW interaction energy for the C270R mutant is mainly contributed by D164, Y268, Q269, R270, H272 and Y273 (Fig. 7b) while the electrostatic energy decreases because of attenuated contributions of E161, D164, E167, and particularly R270 (Fig. 7c and d). The positively charged sidechain of R270 is flexible and is able to approach the P3-Arg residue of the substrate (Fig. S15a†). Unfavored electrostatic interaction results between the protease and substrate as the sidechain of R270 is in proximity to P3-Arg, leading to a decrease in the averaged electrostatic energy in the C270R mutated protease in complex with the substrate (Fig. S15b†). It is noteworthy that most of the other tested mutations in the experimental enzymatic assays influenced the *K*_m_ trivially except C270K and C270Y that also increased the *K*_m_ values.^24^ The influence of C270K on *K*_m_ may follow the same mechanism as C270R but how C270Y increases *K*_m_ needs further studies.

### C270R mutation impeding the C111–H272 proton transfer

**Fig. 8:**
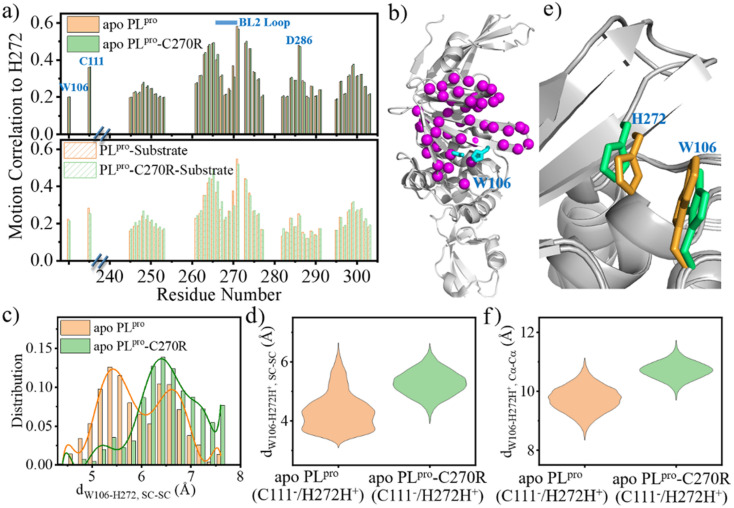
The conformational change of the BL2 loop affects the W106–H272 packing during the proton transfer. (a) Residue–residue motion correlation with H272 in (top) apo and (bottom) substrate-bound wild-type (WT) and C270R mutated SARS-CoV-2 PL^pro^. The data was calculated by using the *cpptraj* module in AMBER for the GaMD simulation trajectories of PL^pro^ systems. To clarify the figure, the residues with low correlation coefficient (*e.g.*, <0.2) in both WT and mutated proteases are not presented. (b) The location of the residues with high motion correlation with H272, as shown by Cα atoms represented as spheres. The catalytic triad is represented by cyan colored sticks. (c) The distribution of the W106–H272 sidechain distance (d_W106–H272,SC–SC_) in apo WT PL^pro^ and C270R mutant carrying the neutral form of the catalytic triad (E_N_). (d) The range of W106–H272H^+^ sidechain distance (d_W106–H272H^+^, SC–SC_) in the IP states of apo WT PL^pro^ and C270R mutant (E_IP_, cation–π interactions considered to be formed upon d_W106–H272H^+^, SC–SC_ < 4.5 Å). (e) The comparison of the configuration of W106 and H272 in the representative structures of the IP states of apo WT PL^pro^ (orange) and C270R mutant (limegreen). (f) The range of W106–H272H^+^ Cα–Cα distance (d_W106–H272H^+^, Cα–Cα_) in apo WT PL^pro^ and C270R mutant.

The change in the structure and/or dynamics of the active site could influence the catalytic reactions as well. Among the three catalytic residues, H272 is the most dynamic one (Fig. 2b) and its motion is mainly correlated with the catalytic partner of C111 and D286 in the palm subdomain, and a W106 residue in the interface between the thumb and palm subdomains (Fig. 8a and b).

**Fig. 9:**
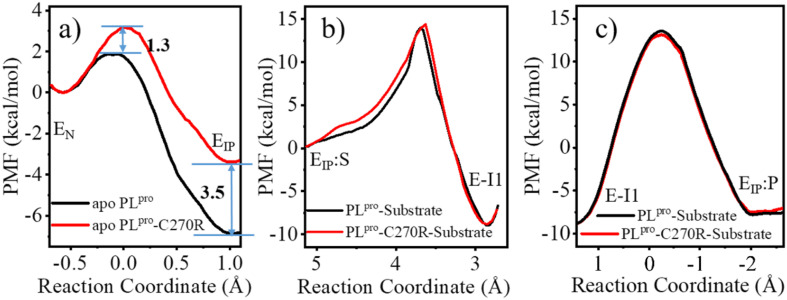
The comparison of the calculated QM/MM free energy profiles associated with (a) C111–H272 proton transfer, (b) acylation, and (c) deacylation reactions for wild-type (WT, black) and C270R mutated (red) SARS-CoV-2 PL^pro^, respectively.

W106 displays a conformation diversity among the crystal structures (Fig. S16†), making it possible to form π–π interactions with the neutral H272 or cation–π interactions with the positively charged H272H^+^ so as to further stabilize the catalytic triad. Such a function could be somehow destroyed by the C270R mutation. As shown in Fig. 8c, the aromatic sidechain of W106 is more likely to approach the imidazole ring of H272 in the apo wild-type than in its C270R mutant in the case of carrying the neutral form of the catalytic triad. And in Fig. 8d, W106 forms steady cation–π interactions with H272H^+^ (d_W106–H272H^+^, SC–SC_ < 4.5 Å) in the apo wild-type protease but not in its C270R mutant when the IP state of the catalytic triad is formed. Accordingly, the QM/MM free energy profile shows that the IP state yielded in the C111–H272 proton transfer is 3.5 kcal mol^−1^ lower and the associated free energy barrier is 1.3 kcal mol^−1^ lower in the apo wild-type PL^pro^ than in its C270R mutant (Fig. 9a). The comparison of the IP states in the two systems indicates that W106 and H272H^+^ have their sidechains at a similar orientation (Fig. 8e) whereas the Cα–Cα distance is larger in the C270R mutant (Fig. 8f). Therefore, the failure of the W106–H272H^+^ cation–π packing in the apo C270R mutant is attributed to the movement of H272 further away from W106 induced by the conformational change of the BL2 loop (Fig. 8e and f).

As the substrate is bound, the ACC group of the substrate is located in the middle between W106 and H272, which can form π–π interactions with W106 and also probably with H272 (Fig. S17a†) in both the wild-type PL^pro^ and C270R mutant. Meanwhile, in the E-I1 intermediates of both complexes, the W106 aromatic sidechain is not in proximity to H272 (Fig. S17b†). The C270R induced impact on H272 in apo PL^pro^ thus cannot be seen in the substrate-bound complex. On the other hand, H272 accepts the proton from C111 in the C111–H272 proton transfer, donates its Nδ-H proton to the substrate P1-N atom in the acylation step, and accepts the proton from the deacylating water in the deacylation step, respectively. As indicated by the respective reaction paths of these steps, the hydrogen binding to H272-Nδ in the C111–H272 proton transfer step (Fig. S18a and d†) and the hydrogen breaking from H272-Nδ-H in the acylation step (Fig. S18b and e†) are involved in the respective transition states whereas the hydrogen bond to H272-Nδ is already formed before the transition state of the deacylation (Fig. S18c and f†). This implies less distinguished importance of H272 in the deacylation step compared to the C111–H272 proton transfer and acylation. Taken together, the C270R mutation induced change mainly impacts the C111–H272 proton transfer step in the apo enzyme but not substrate-involved acylation and deacylation.

Accordingly, the comparison of the free energy profiles associated with the acylation and deacylation reactions shows that the C270R mutant shares very similar features with the wild-type PL^pro^, and no obvious difference in the free energy barriers can be seen between the wild-type and C270R mutated PL^pro^ (Fig. 9b and c). Meanwhile, the corresponding reaction paths are almost identical for the wild-type (Fig. S18e and f†) and C270R mutated PL^pro^ (Fig. S18h and i†). For instance, in the acylation, the proton transfer from H272H^+^ to the P1′-N atom precedes the formation of the C111-Sγ–P1-C bond as well as the associated breakage of the P1-C–P1′-N bond. In the deacylation, the proton transfer from the water molecule to H272 precedes the binding of the water oxygen to the P1-C atom and the breakage of the C111-Sγ–P1-C bond, and the latter two events occur concertedly.

The C270 or R270 sidechain almost always stays away from H272 (>5.0 Å) in the MD simulations (Fig. S19†) and the QM/MM MD simulations of proton transfer (Fig. S20†). Therefore, the C270R mutation exerts an inhibitory influence on the proton transfer reaction in an indirect manner. Additionally, the change in the associated free energy profiles should not be the artifact caused by measurement uncertainty. The QM/MM MD simulations with various initial structures were tested that always obtained similar PMF profiles (Fig. S5†). The error bars derived with 100 ps/block averaging in individual umbrella sampling windows were also calculated to evaluate the convergence of QM/MM MD simulations. Fig. S21† shows that for the tested systems, the error bars are quite small compared to the detailed free energy values, suggesting the convergence of the relevant simulations.

It is also noteworthy that the deacylation step in path I involving a proton transfer from H272H^+^ to C111^−^ was also simulated for the C270R mutant as a control. As shown in Fig. S22,† the deacylation with the regeneration of the neutral form (C111/H272) of the catalytic triad is still highly endergonic with a higher free energy level of E_N_:P relative to E-I1 (14.4 kcal mol^−1^), suggesting again that the deacylation of SARS-CoV-2 PL^pro^ regenerates the ion-paired catalytic triad.

### An assumption on how the C270R mutation affects the proteolysis

The Michaelis–Menten equation for the simplest model of enzyme kinetics (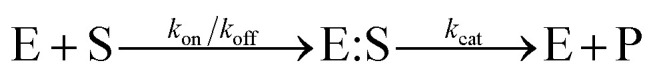) is: 
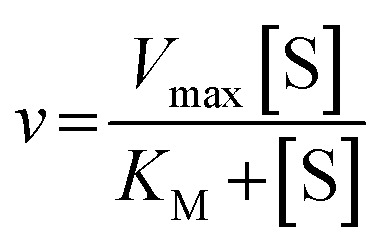
, where *V*_max_ = *k*_cat_[*E*_T_] and 
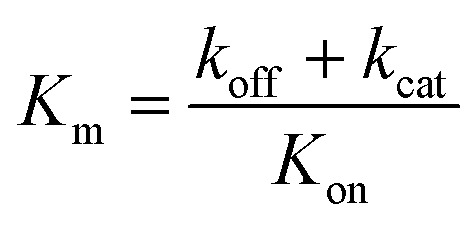
 (*k*_on_ and *k*_off_ are the association and dissociation rate constants of the enzyme-substrate binding/unbinding; *k*_cat_ is a first-order rate constant to give free enzyme E and product P; [S] is the concentration of the substrate S; [*E*_T_] is the total enzyme concentration).^59^ The PL^pro^ proteolysis is more complex, including not only the enzyme catalyzed acylation and deacylation reactions but also the prerequisite enzyme proton transfer that yields E_IP_ for the proteolysis:1
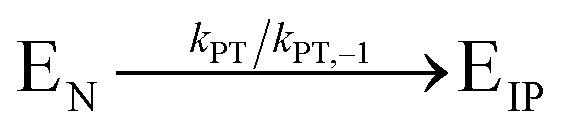
2
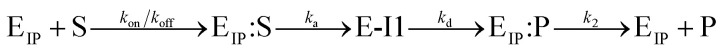
where *k*_PT_ and *k*_PT,−1_ are the rate constants of the C111–H272 proton transfer and reverse step; *k*_a_ and *k*_d_ are the rate constants of the acylation and deacylation steps, and *k*_2_ the dissociation rate constant of the enzyme-product complex, respectively.

From the steady-state approximation,^60^*V*_max_ and *K*_m_ can be represented as:3
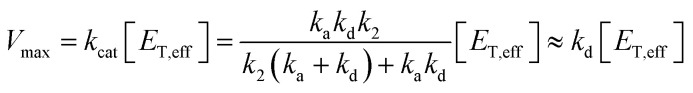
4
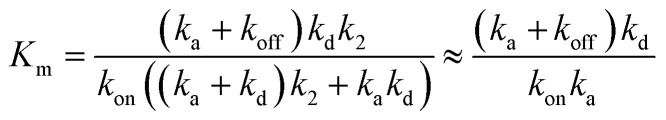


Only the ion-paired enzyme is involved in the PL^pro^ proteolysis. Thus, the total concentration of the effective enzyme in eqn (3) should be: [*E*_T,eff_] = [E_IP_] + [E_IP_:S] + [E-I1] + [E_IP_:P]. The C270R mutation studied here does not affect *k*_d_, the rate constant of the rate-limiting step (deacylation) of SARS-CoV-2 PL^pro^, but rather increases the free energy barrier and particularly the free energy level of the ion-paired catalytic triad and thus makes it more difficult for the C111–H272 proton transfer to occur. Meanwhile, the C270R mutation also decreases the PL^pro^-substrate binding interaction strength, making the substrate binding more difficult. Together, the C270R mutation might reduce [*E*_T,eff_] in eqn (3) as compared to the wild-type protease, thus displaying an inhibitory effect on the catalytic activity of SARS-CoV-2 PL^pro^.

One should note that although the enzymatic assay experimentally showed the inhibitory effect of the C270R mutant, the *V*_max_ ratio of the mutant to the wild-type PL^pro^ is not large (0.32-fold).^24^ Accordingly, if the *V*_max_ is solely influenced by *k*_d_ (eqn (3)), then the detailed free energy barrier increase should also not be large (∼0.68 kcal mol^−1^), and accurate measurement should be very difficult using the current simulation methodologies.

## Conclusions

In this work, we performed a comprehensive *in silico* study on the catalytic mechanism of the wild-type and C270R mutant of SARS-CoV-2 PL^pro^, and tried to explain how the mutation on C270 exerts an influence on the catalytic activity. Combined with ∼20 μs MD simulations, the MM/PBSA measurements revealed that the C270R mutation weakens the protease-substrate binding strength, consistent with the experimentally observed higher Michaelis constant (*K*_m_) value of this mutant compared to the wild-type SARS-CoV-2 PL^pro^.^24^ And the QM/MM MD calculations showed that the C270R mutation to some extent increases the free energy barrier of the C111–H272 proton transfer and the free energy level of the ion-paired catalytic triad, probably linked to the experimentally observed inhibition of C270R on the catalytic activity. Additionally, the present simulations also displayed consistencies with previous theoretical studies. Two previous QM/MM MD simulations on SARS-CoV-2 3CL^pro^ were used for comparison.^25,26^ The present studies had a similar free energy barrier for the acylation step to that in ref. 26 (13.6 kcal mol^−1^*vs.* 14.6 kcal mol^−1^) and a similar free energy barrier for the deacylation step to that in ref. 25 (22.4 kcal mol^−1^*vs.* 22.8 kcal mol^−1^), with the two literatures sharing a similar acylation and deacylation mechanism, respectively, to the one investigated in the present study.

It is observed that in comparison to SARS-CoV-2 3CL^pro^,^26,42,61^ the tight contact of D286 with H272 facilitating the deprotonation of C111 in PL^pro^ decreases the free energy barrier of the C111–H272 proton transfer and lowers the free energy level of the ion pair (E_IP_) relative to the neutral form (E_N_) in apo PL^pro^. These results suggest that the catalytic triad prefers the state containing the ion pair (C111^−^/H272H^+^) in apo SARS-CoV-2 PL^pro^. Therefore, while the proton transfer mechanism in apo and substrate-bound enzymes is still under debate for SARS-CoV-2 or SARS-CoV 3CL^pro^,^25,26,36–43^ the proton transfer mechanism of SARS-CoV-2 PL^pro^ is more definite. Following path II in Fig. 1, the substrate binds to PL^pro^ containing the ion-paired catalytic triad, carries out the acylation and deacylation step by step, and recovers the catalytic triad to the ion pair state for the next catalytic cycle.

The sidechain dynamics of H272 is highly correlated with a series of residues including the BL2 loop (G266–G271) and W106. The C270R mutation alters the conformation of the BL2 loop, enlarges the W106–H272 distance and thus hinders the packing of W106 to H272 in apo protease, impairing the function of H272 as a general acid–base in proton transfer. The binding of the substrate fills the S1′ subsite, which, however, blocks the interaction from W106 to H272, impairing the influence of C270R mutation on H272. As result, the influence of the C270 mutation is mainly displayed in the proton transfer reaction in apo PL^pro^ but not substrate-involved acylation and deacylation. An assumption is ultimately proposed that the C270R mutation impedes the C111–H272 proton transfer and meanwhile weakens the PL^pro^-substrate binding, decreasing the concentration of the effective enzyme (eqn (3)) and thus exerting an inhibitory effect on the catalytic activity of SARS-CoV-2 PL^pro^. These results afford key clues for the future development of C270-target covalent inhibitors with greatly improved inhibitory activity by enlarging the difference in *V*_max_.

## Computational methods

### Simulation systems setup

The structural dynamics of the apo SARS-CoV-2 PL^pro^ (wild-type) and C270R mutant, and their respective complexes with the substrate were measured by using Gaussian accelerated MD (GaMD), a sophisticated enhanced sampling MD simulation method which has been extensively utilized in a variety of biomolecular simulations for protein folding, protein conformational transition, and protein–ligand binding.^45,46,62–66^ Detailed methodology descriptions of GaMD can be found in previous literatures.^45,46^

The atomic coordinates of apo SARS-CoV-2 PL^pro^ were retrieved from the Protein Data Bank (PDB code 6WRH) with a resolution of 1.6 Å.^20^ The C111S mutant was recovered, all crystal water was maintained, and the solvated molecules (phosphate, glycerol, and chloride ions) were removed. Meanwhile, the substrate was docked into PL^pro^ through aligning the ISG15 complexed system (PDB code 6YVA)^6^ to 6WRH and only the RLRGG segment was maintained from the ISG15 protein as the substrate used for the simulation. The protein termini remained freely charged (uncapped) whereas the N- and C-termini of the substrate peptide RLRGG were capped with the acetyl (Ac) group and the 7-amino-4-carbamoylmethylcoumarin (ACC) fluorophore (Fig. S1†), respectively. ACC has often been used as a fluorescent tag in enzymatic assay.

The protonation states of all titratable residues at pH 7.5 were evaluated using Schrodinger suite software. All residues were found in their standard protonation state. After a detailed inspection of the environment surrounding each histidine residue, all histidines were neutral except that His17 was positively charged. His47, His73, His89, His175 and His275 were protonated in the Nδ position, while the remaining His50, His272, and His255 were protonated on Nε. No S–S linkage was detected between Cys residues. All cysteine residues except Cys189, Cys192, Cys224 and Cys226 that form coordinate bonds with Zn^2+^ were protonated. The C270 was manually replaced with arginine in the mutant system.

Each protein (and substrate) system was solvated in a cubic box filled with a total of ∼18 000 water molecules, in which multiple Na^+^/Cl^−^ ions were added to neutralize the protein charges. The AMBER 18 suite of programs^67^ was employed for simulations with the underlying force fields of FF14SB force field^68^ for protein and TIP3P model^69^ for water molecules. The coordinate bond between Zn^2+^ and the surrounding cysteines in the zinc fingers subdomain of PL^pro^ was modeled using the *MCPB.py* program implemented in AMBER 18.^70^ The ACC group and the non-standard residues in the peptide intermediates (*e.g.*, E-I1, E-I2) were modeled using a generalized AMBER force field (GAFF)^71^ with restrained electrostatic potential (RESP)^72^ partial charges fitted with Gaussian 09.^73^

### Molecular dynamics simulations

Each constructed system as mentioned above was initially minimized for 50 000 steps and heated to 300 K, with the protein (and substrate) heavy atoms being fixed using a harmonic restraint with a force constant of 10.0 kcal mol^−1^ Å^−2^. Subsequently, the protein was relaxed by two steps of equilibrium at a constant temperature of 300 K and constant pressure of 1 atm (NPT ensemble): 2 ns for relaxing the protein sidechain and 2 ns for the protein main chain. The SHAKE algorithm was used to fix all covalent bonds involving hydrogen atoms and periodic boundary conditions were used to avoid edge effects.^74^ The Particle Mesh Ewald method was applied to treat long-range electrostatic interactions and the cutoff distance for long-range terms (electrostatic and van der Waals (vdW) energies) was set as 10.0 Å.^75^ The Langevin dynamics with a collision frequency of 2.0 ps^−1^ was adopted to control the temperature. Finally, the GaMD simulations were performed on the equilibrated system using the GaMD module implemented in the GPU version of AMBER 18, including a 10 ns short conventional MD simulation for collecting the potential statistics to define the GaMD acceleration parameter values, a 10 ns equilibration after adding the boost potential, and finally ∼2 μs GaMD production simulation with randomized initial atomic velocities.

All GaMD simulations were run at the “dual-boost” level by setting the reference energy to the lower bound, one boost potential being applied to the total potential and the other to the dihedral energetic term. The average and the standard deviation (SD) of the system potential energies were calculated every 250 000 steps (0.5 ns). The upper limit of the boost potential SD was set to 6.0 kcal mol^−1^ for both the dihedral and the total potential energetic terms. The coordinates were saved every 10 000 steps.

To sample the existing probability of a water molecule in between H272 and the P1-C atom of the substrate for the deacylation process, a conventional MD simulation was performed lasting 2 μs for each acyl-enzyme complex system.

### Molecular mechanics Poisson Boltzmann surface area (MM/PBSA) calculation

To assess the PL^pro^-substrate binding interactions and the effects from the mutation on the C270 residue, the most populated structure of each PL^pro^-substrate complex system was identified from the GaMD simulation trajectory through the clustering analysis using the MMTSB toolset.^76^ The identified complex structure was then solvated, minimized, heated up, and equilibrated following the same procedure as in the GaMD simulation. An additional 10 ns equilibrium simulation was run by fixing the protein and substrate Cα atoms with a harmonic force constant of 1.0 kcal mol^−1^ Å^−2^. The trajectory was analyzed by the standard approach of MM/PBSA calculation using the *MMPBSA.py.MPI* program^77^ in AMBER software. The vdW and electrostatic interaction energies between individual protein residues and the substrate were extracted for data analysis, through a per-residue decomposition type that calculates the energy contribution of single residues by summing its interactions over all substrate atoms. This functionality was fulfilled using the *sander.MPI* program in AMBER.

### Quantum mechanics/molecular mechanics (QM/MM) MD simulation

Exploration of the free energy profiles associated with the fundamental steps of the cysteine–histidine ion pair formation, acylation and deacylation has been carried out using QM/MM MD simulations. In all simulations, the sidechains of the catalytic triad (C111, H272 and D286) and a fragment of the peptide substrate were involved in the QM region, while the remaining part of the system was described at the MM level. Specifically, the QM-treated fragment of the substrate included the P1′-ACC group, P1-Gly, and the peptide bonds up to the Cα atoms of P2-Gly. In the hydrolysis step of the acyl-enzyme, a water molecule was also included in the QM region. As the QM region crossed covalent bonds, the QM/MM boundary was chosen to cut C–C non-polar bonds and link atoms (hydrogens) were added automatically for the QM calculation without user intervention.^78,79^ Using the acylation reaction as an example, the QM/MM boundary was set to cut the Cα–Cβ bonds of enzyme residues (C111, H272 and D286), respectively, and the C–Cα bond of the substrate P2-Gly residue. A density functional theory-based tight-binding (DFTB)^80^ was used to describe the QM subsystem, a method that has been extensively shown to provide fairly reliable structures and energies in agreement with experimental findings with accelerated calculation speed as compared to traditional DFT methods, which makes it attractive for free energy simulations of biomolecular systems.^81–85^ All calculations were run using the CPU implementation of the *sander.MPI* program in AMBER 18.^67^ A cutoff radius of 12 Å was used for QM/MM interactions and the temperature was controlled at 300 K.

The reaction pathway was obtained using steered molecular dynamics (SMD)^86^ in the QM region following specific reaction coordinates (see the detailed description of the reaction coordinates in the “Results and discussion” section). Multiple harmonic force constants within 100 ∼ 500 kcal mol^−1^ Å^−2^ were tested to pull the system along the predefined reaction coordinate to see whether the yielded traction pathways are converged or not. Then the detailed free energy profile was calculated, in terms of potentials of mean force (PMF), for every step of the reaction using the Umbrella Sampling (US) approach^87^ combined with the Weighted Histogram Analysis Method (WHAM).^88^ Series of QM/MM US simulations were performed adding a constraint along the predefined reaction coordinates with an umbrella force constant of 100 kcal mol^−1^Å^−2^. Structures selected from the SMD simulation were used as starting points for the US simulations. The detailed number of QM/MM US windows for all systems is shown in Table 1, making sure the sampled reaction path is overlapped between individual windows. In every window, simulation was performed with a total of 250 ps of equilibration and 250 ps of production at 300 K with a time step of 1 fs.

## Data availability

The data that support the findings of this study are available in the ESI of this article.†

## Author contributions

Y. X. conceived and designed the study. Q. S. conducted all the theoretical modeling and simulations. Q. S. and Y. X. wrote the initial manuscript. All authors contributed to the data analysis and the revisions of the manuscript, and approved the final version.

## Conflicts of interest

There are no conflicts to declare.

## Supplementary Material

SC-014-D3SC00166K-s001
