# Supplementary material for: Unraveling the catalytic mechanism of SARS-CoV-2 papain-like protease with allosteric modulation of C270 mutation using multiscale computational approaches
Source: Chem Sci. 2023 Apr 11;14(18):4681–96. doi: 10.1039/d3sc00166k (PMC10171076; doi:10.1039/d3sc00166k)
Supplement: SC-014-D3SC00166K-s001 [file SC-014-D3SC00166K-s001.pdf]

Electronic Supplementary Material (ESI) for Chemical Science.
This journal is © The Royal Society of Chemistry 2023

Supplementary Information

Unraveling the Catalytic Mechanism of SARS-CoV-2 Papain-like Protease with Allosteric Modulation of C270 Mutation Using Multiscale Computational Approaches

Qiang Shao[a,b*], Muya Xiong[a,b], Jiameng Li[c], Hangchen Hu[d], Haixia Su[a], Yechun Xu[a,b,c,d*]

[a]CAS Key Laboratory of Receptor Research and State Key Laboratory of Drug Research, Shanghai Institute of Materia Medica, Chinese Academy of Sciences, Shanghai 201203, China

[b]University of Chinese Academy of Sciences, Beijing 100049, China

[c]School of Chinese Materia Medica, Nanjing University of Chinese Medicine, Nanjing 210023, China

[d]School of Pharmaceutical Science and Technology, Hangzhou Institute for Advanced Study, University of Chinese Academy of Sciences, Hangzhou 310024, China

*Corresponding Email: qshao@simm.ac.cn (Q. Shao); ycxu@simm.ac.cn (Y. Xu)

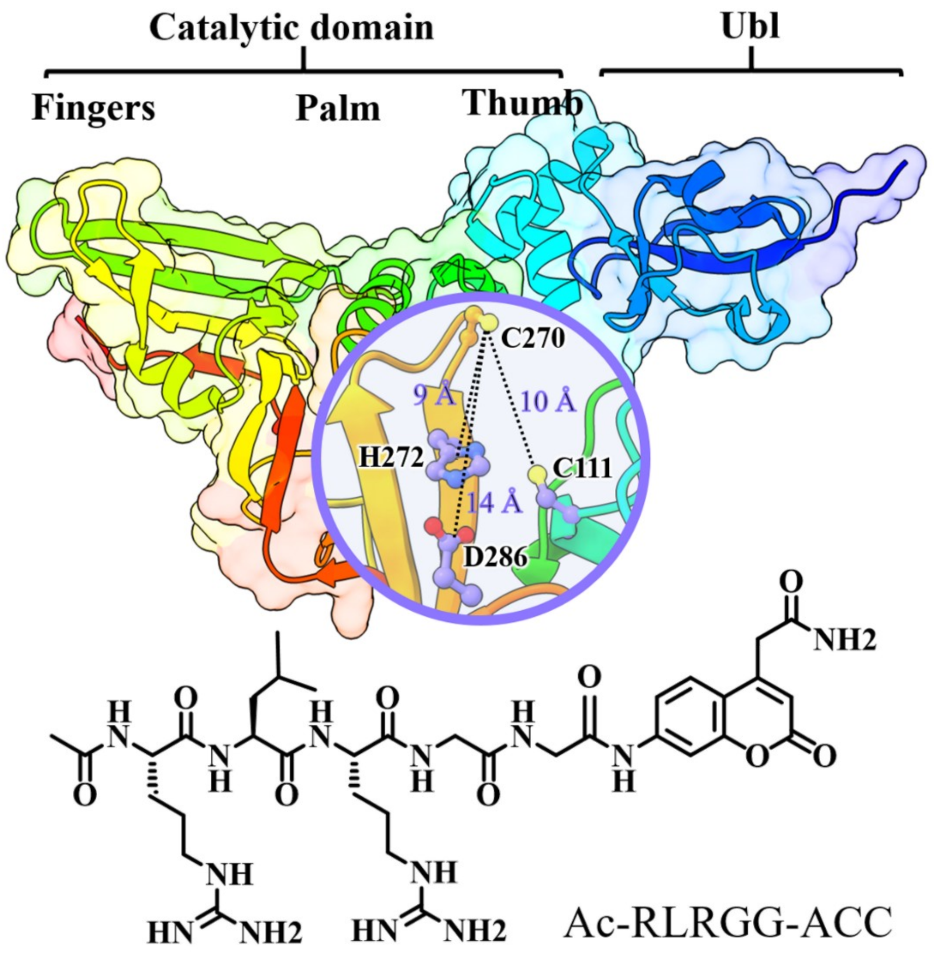

Fig. S1 Structures of SARS-CoV-2 PL^{pro} and the pentapeptide substrate studied in the present study. An expanded view is provided for C270 at BL2 loop and the catalytic triad C111-H272-D286. The dash lines represent sidechain distances between C270 and three catalytic residues.

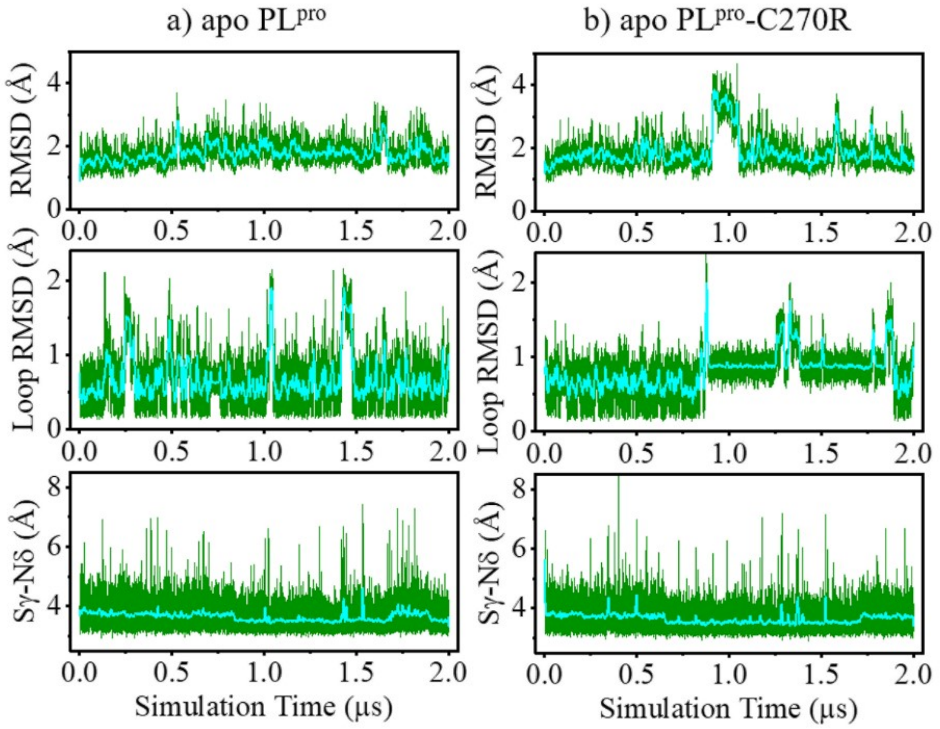

Fig. S2 GaMD simulation trajectories of apo structures of (a) the wild-type SARS-CoV-2 PL^{pro} (apo PL^{pro}) and (b) C270R mutant (apo PL^{pro}-C270R) as indicated by the time series of the main chain root-mean-square deviations (RMSDs) of protein and BL2 loop, and the distance between C111-Sγ and H272-Nδ atoms (Sγ-Nδ), respectively (from top to bottom).

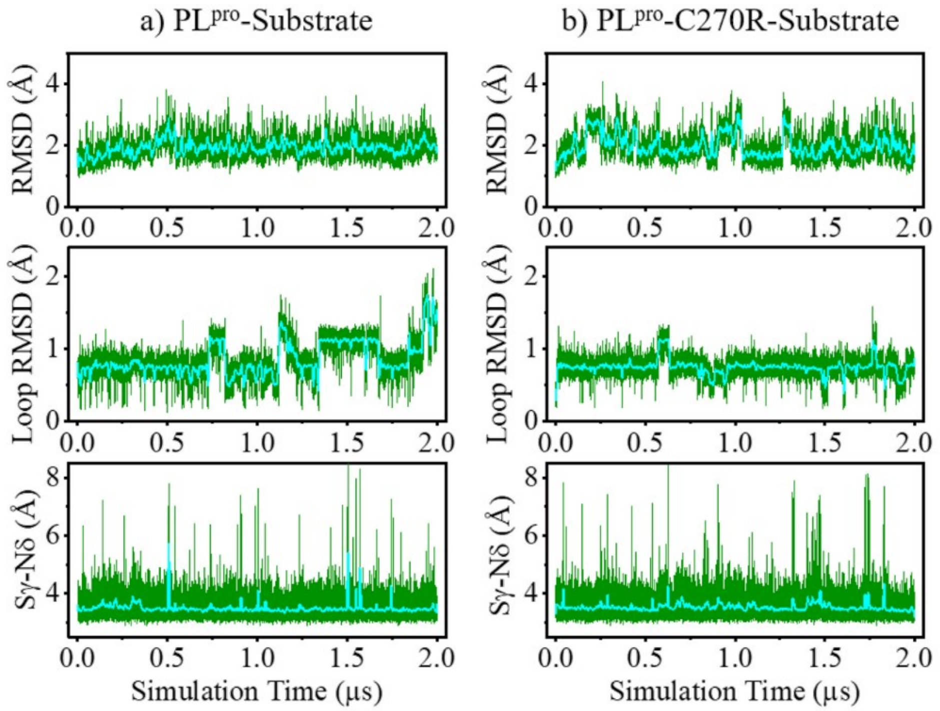

Fig. S3 GaMD simulation trajectories of the substrate (Ac-RLRGG-ACC)-bound structures of (a) the wild-type SARS-CoV-2 PL^{pro} (PL^{pro}-Substrate) and (b) C270R mutant (PL^{pro}-C270R-Substrate) as indicated by the time series of the main chain RMSDs of protein and BL2 loop, and the distance between C111-Sγ and H272-Nδ atoms, respectively (from top to bottom).

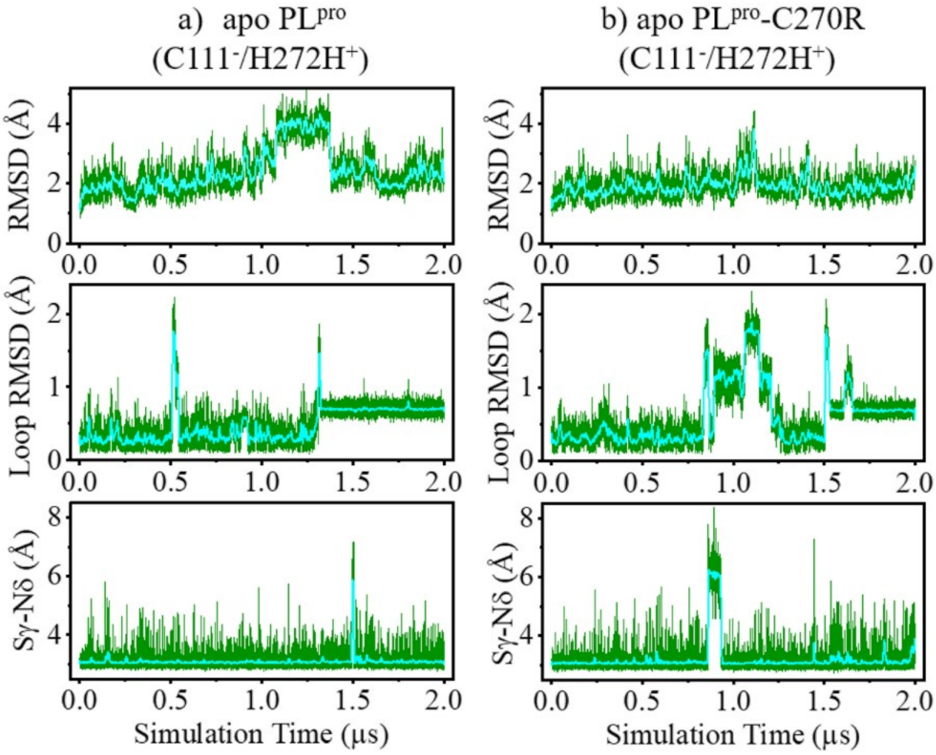

Fig. S4 GaMD simulation trajectories of apo structures of (a) the wild-type SARS-CoV-2 PL^{pro} and (b) C270R mutant containing ion pair form of C111^{-}/H272H^{+}. The trajectories are represented by the time series of the main chain RMSDs of protein and BL2 loop, and the distance between C111-Sγ and H272-Nδ atoms, respectively (from top to bottom).

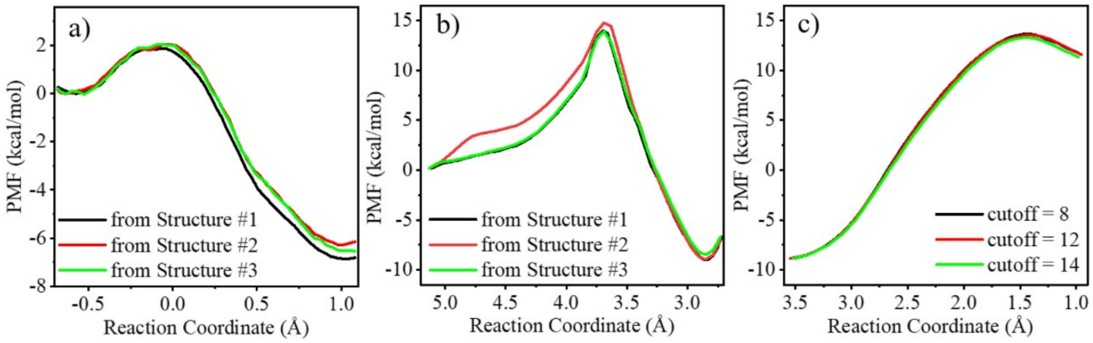

Fig. S5 The evaluation of simulation factors affecting QM/MM results. (a-b) QM/MM free energy profiles associated with (a) the C111–H272 proton transfer in apo wild-type SARS-CoV-2 PL^{pro} and (b) the acylation reaction in substrate-bound wild-type PL^{pro} using different initial structures. (c) QM/MM free energy profile associated with the E-I1 → E-I2 substep of deacylation reaction in substrate-bound wild-type SARS-CoV-2 PL^{pro} calculated using multiple cutoff values.

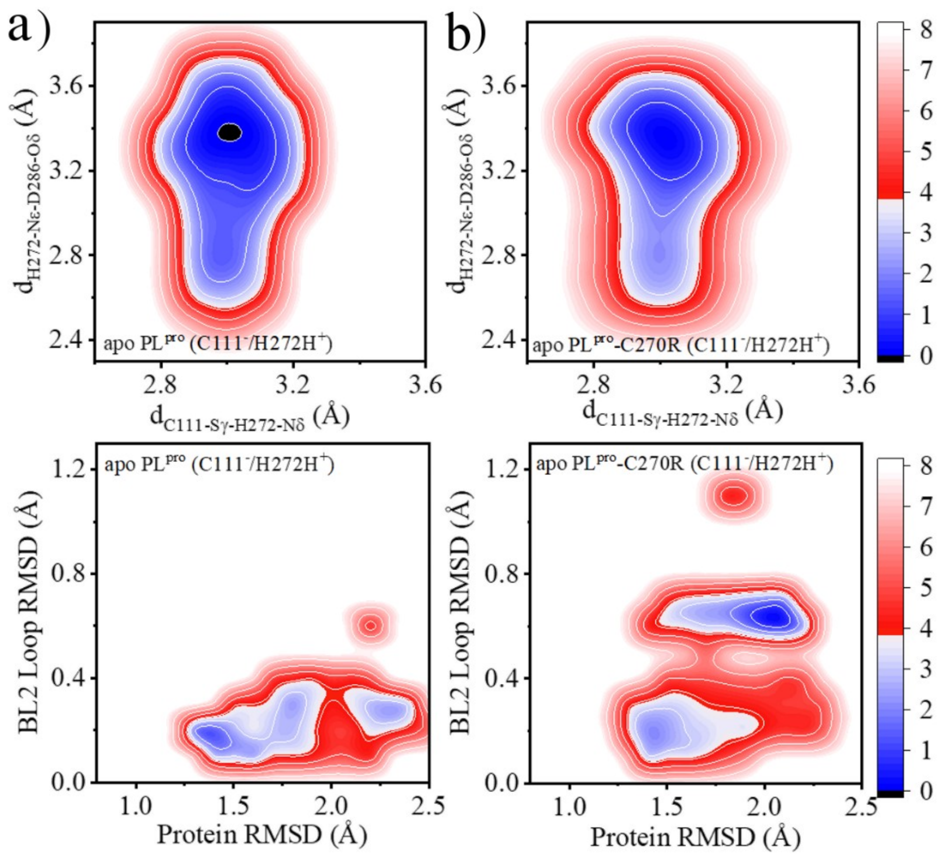

Fig. S6 Free energy landscapes (FELs) along the collective variables of the distance between C111-Sγ and H272-Nδ ($d_{C111\text{-}S\gamma\text{–}H272\text{-}N\delta}$) and the distance between H272-Nε and D286-Oδ ($d_{H272\text{-}N\varepsilon\text{–}D286\text{-}O\delta}$), the main chain RMSDs of protein and BL2 loop for GaMD simulations of apo (a) wild-type SARS-CoV-2 PL^{pro} and (b) C270R mutant containing ion pair form of C111^{-}/H272H^{+}. The contours in the two-dimensional subspace are spaced at intervals of 1.0 kcal/mol.

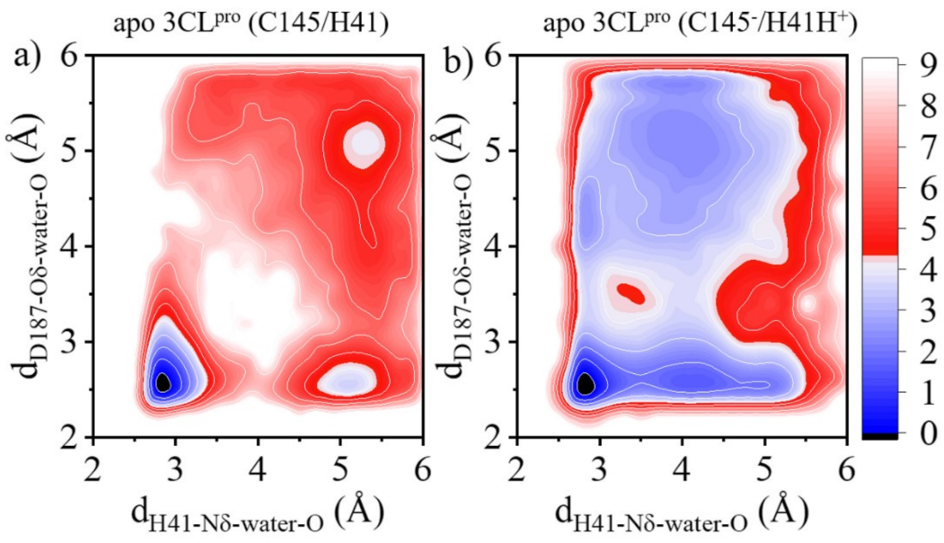

Fig. S7 FELs along the distance between H41-Nδ and the oxygen of the crystal water ($d_{H272\text{-}N\delta\text{–}water\text{-}O}$) and the distance between D187-Oδ and the oxygen of the crystal water ($d_{D286\text{-}O\delta\text{–}water\text{-}O}$) for conventional MD simulations of apo SARS-CoV-2 3CL^{pro} containing either (a) the neutral form or (b) ion pair of the catalytic H41 and C145. The contours in the two-dimensional subspace are spaced at intervals of 1.0 kcal/mol.

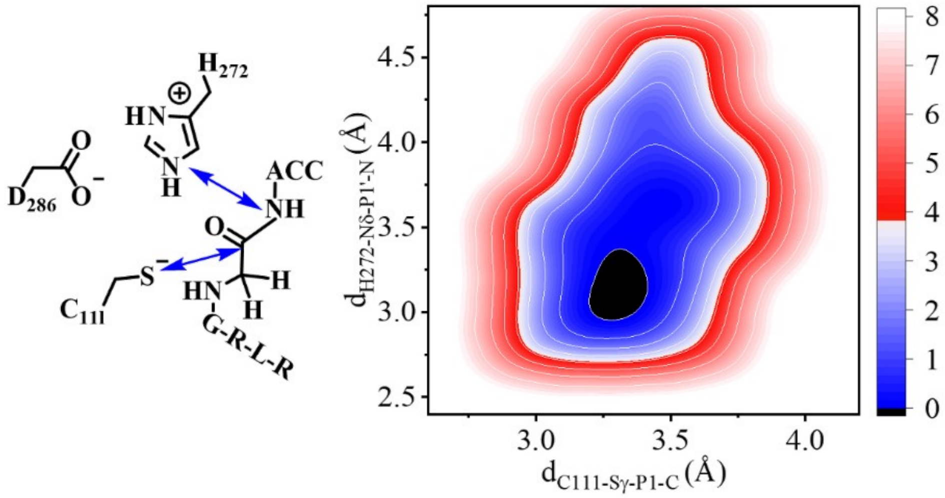

Fig. S8 FELs along the distance between C111-Sγ and P1-C atoms, and the distance between H272-Nδ and P1′-N atoms for the GaMD simulation of substrate-bound wild-type SARS-CoV-2 PL^{pro}. The contours in the two-dimensional subspace are spaced at intervals of 1.0 kcal/mol.

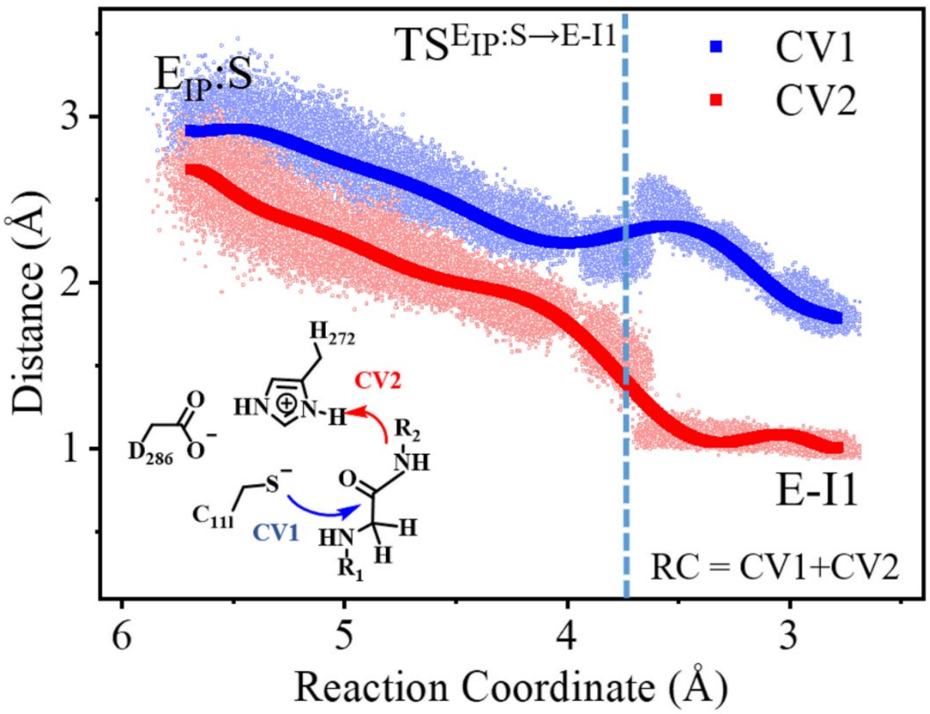

Fig. S9 Evolution of the distances selected as the collective variables (CVs) along the reaction coordinate of the acylation transition (E_{IP}:S → E-I1) in SARS-CoV-2 PL^{pro}. CV1: $d_{C111\text{-}S\gamma–P1\text{-}C}$ (the distance between C111-Sγ and P1-C atoms), CV2: $d_{H272\text{-}H\delta–P1'\text{-}N}$ (the distance between H272-Hδ and P1′-N atoms). Dash line represents the position of the transition state.

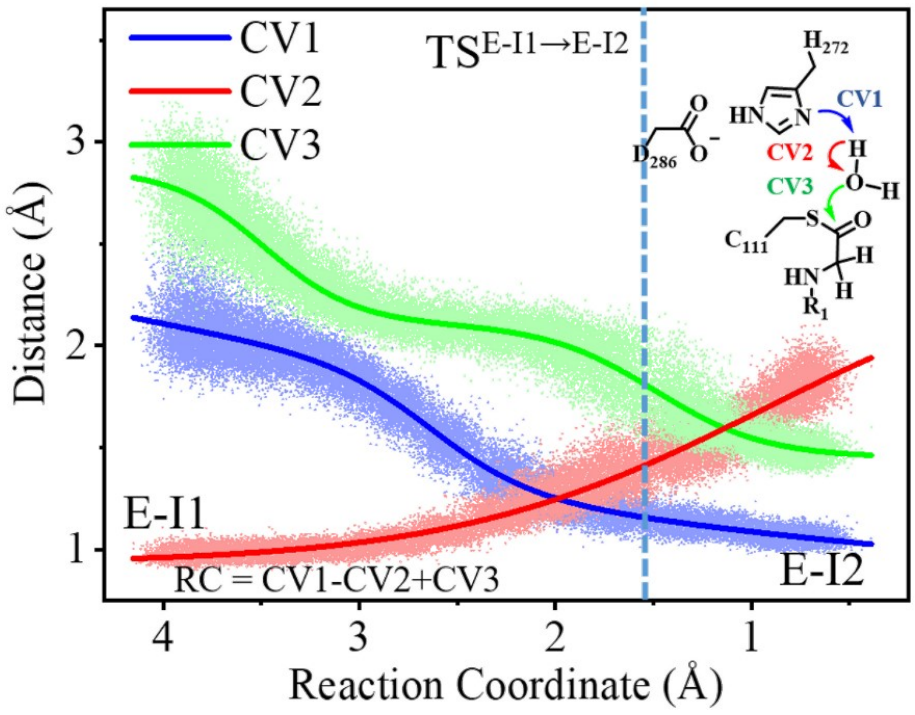

Fig. S10 Evolution of the distances selected as the collective variables (CVs) along the reaction coordinate of the E-I1 → E-I2 substep of the deacylation reaction in path I. CV1: $d_{H272\text{-}N\delta\text{–}water\text{-}H}$ (the distance between H272-Nδ atom and the hydrogen of deacylating water), CV2: $d_{water\text{-}O\text{–}water\text{-}H}$ (the distance between the oxygen and hydrogen atoms of deacylating water), CV3: $d_{water\text{-}O\text{–}P1\text{-}C}$ (the distance between the oxygen of deacylating water and P1-C). Dash line represents the position of the transition state.

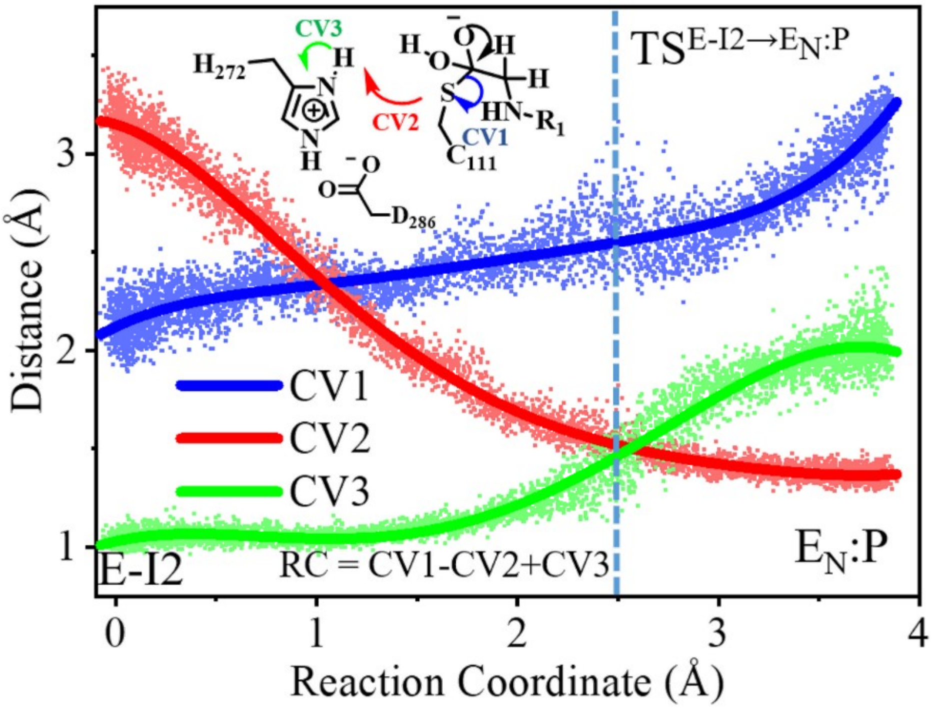

Fig. S11 Evolution of the distances selected as the collective variables (CVs) along the reaction coordinate of the E-I2 → E_N:P substep of the deacylation reaction in path I. CV1: $d_{C111\text{-}S\gamma–P1\text{-}C}$ (the distance between C111-Sγ and P1-C atoms), CV2: $d_{C111\text{-}S\gamma–H272\text{-}H\delta}$ (the distance between C111-Sγ and H272-Hδ atoms), CV3: $d_{H272\text{-}H\delta–H272\text{-}N\delta}$ (the distance between H272-Hδ and H272-Nδ atoms). Dash line represents the position of the transition state.

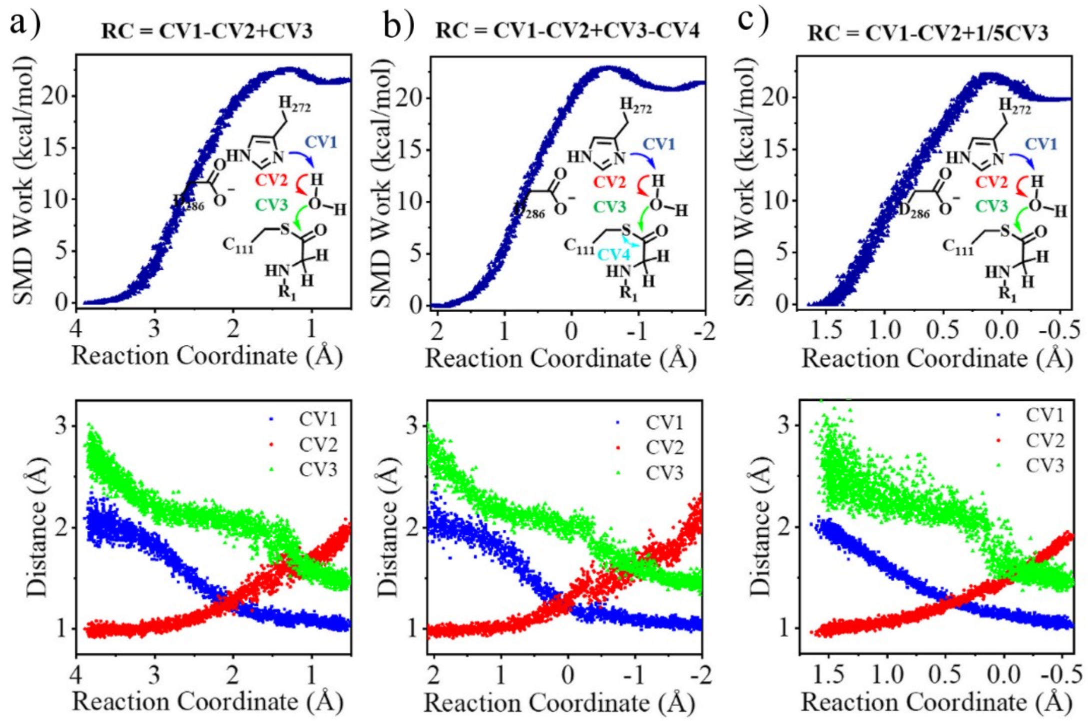

Fig. S12 The QM/MM SMD work applied for the completion of the E-I1 → E-I2 substep of the deacylation transition in path I along multiple predefined reaction coordinates, and the evolution of the distances selected as the collective variables (CVs) along the corresponding reaction coordinates.

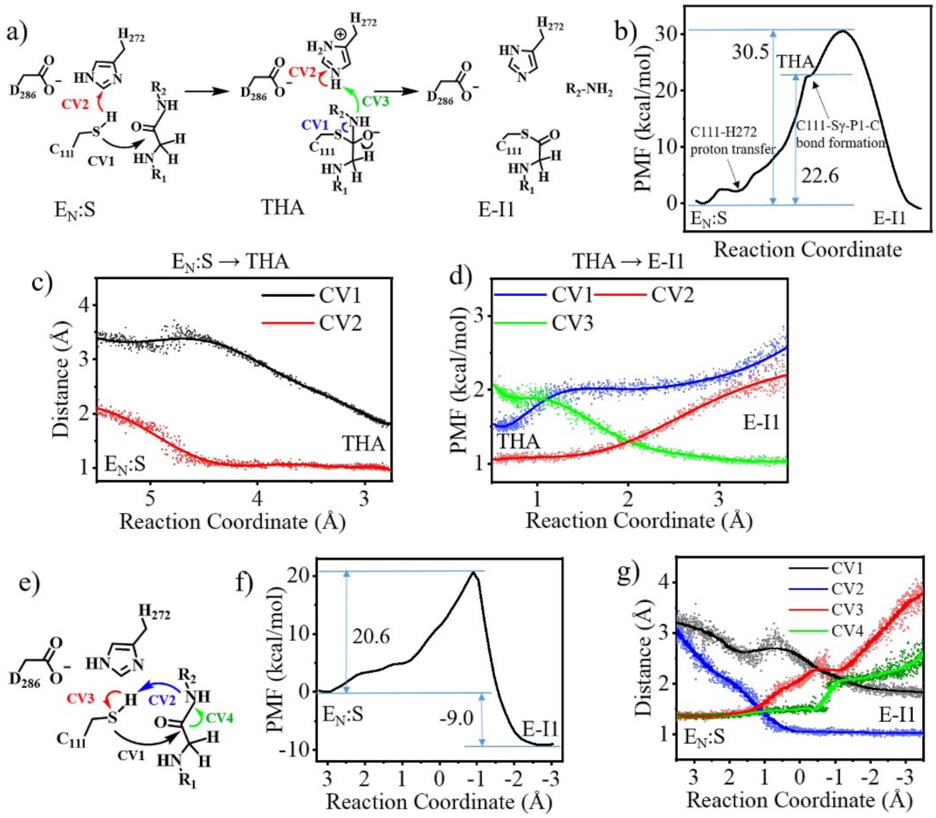

Fig. S13 (a) Scheme indicating a possible molecular mechanism of acylation with a concerted process of C111-Sγ attack to the substrate P1-C and the C111–H272 proton transfer. In this case, the acylation is proceeded by two substeps of E_N:S → THA and THA → E-I1. THA is a transient thiohemiketal state. (b) The QM/MM free energy profile associated with the acylation as described in (a). In the profile, the arrows show the positions where the C111–H272 proton transfer and the C111-Sγ–P1-C bond formation occur. (c-d) Evolution of the selected collective variables (CVs) in the two substeps of acylation, respectively. In the E_N:S → THA substep, the reaction coordinate is defined as RC = CV1 + CV2. CV1: the distance between C111-Sγ and P1-C atoms, CV2: the distance between C111-Hγ and H272-Nδ atoms. In the THA → E-I1 substep, the reaction coordinate is defined as RC = CV1 + CV2 - CV3. CV1: the distance

between P1-C and P1′-N atoms, CV2: the distance between H272-Nδ and H272-Hδ atoms, CV3: the distance between P1′-N and H272-Hδ.

(e) Scheme indicating a possible molecular mechanism of acylation with a concerted process of C111-Sγ attack to the substrate P1-C and the proton transfer from C111-Hγ to P1′-N. H272 is not involved in such a concerted acylation reaction between C111 and substrate. (f) The QM/MM free energy profile associated with the acylation as described in (e), with the reaction coordinate defined as RC = CV1 + CV2 - CV3 - CV4. CV1: the distance between C111-Sγ and P1-C atoms, CV2: the distance between P1′-N and C111-Hγ atoms, CV3: the distance between C111-Sγ and C111-Hγ atoms, CV4: the distance between P1-C and P1′-N atoms. (g) Evolution of the CVs along the reaction coordinate.

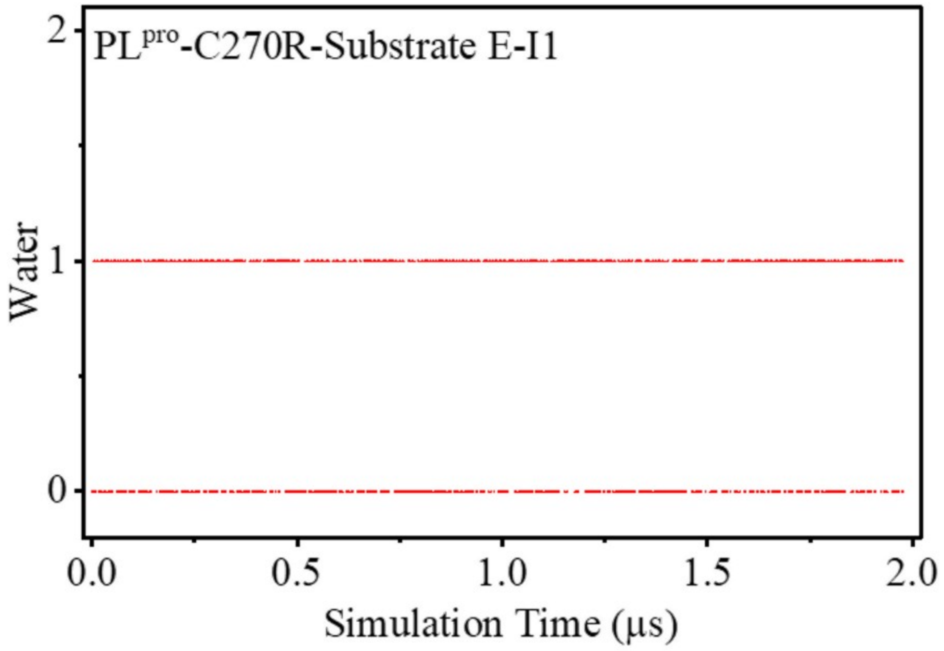

Fig. S14 A cMD simulation trajectory indicating the presence of water in between H272 and the carbonyl carbon of the substrate that is ready for the deacylation reaction for the substrate-bound PL^{pro} C270R mutant system.

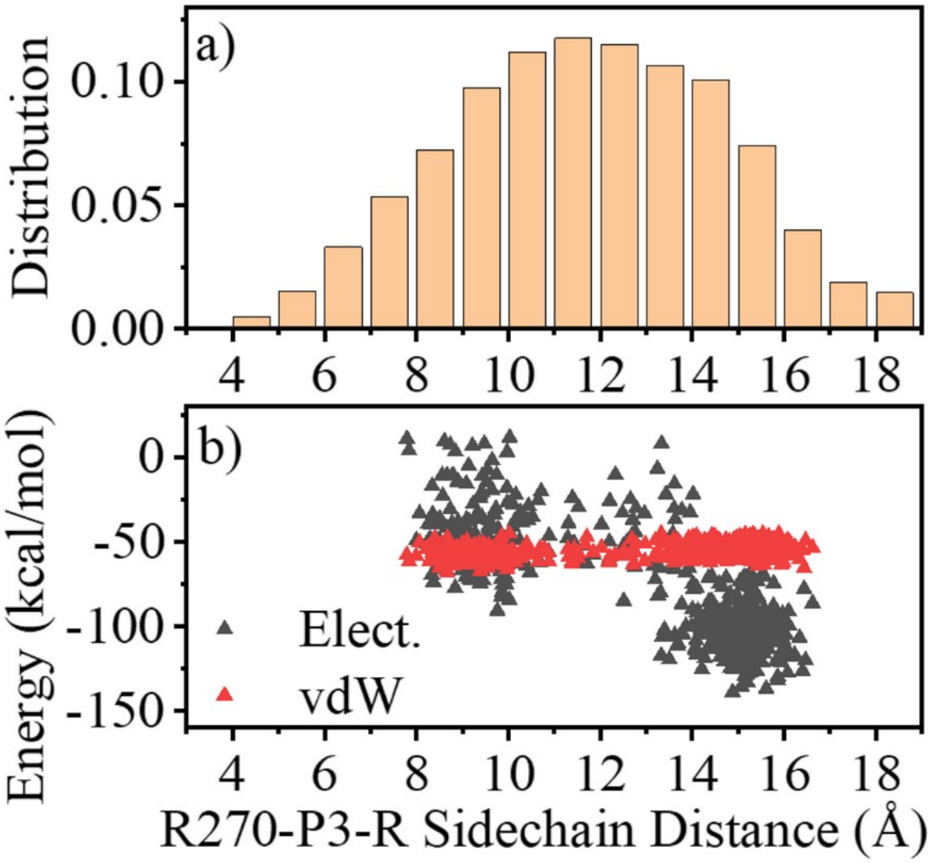

Fig. S15 (a) The distribution of the sidechain distance between PL^{pro} R270 and the arginine at the P3 site (P3-R) of the substrate in the GaMD simulation of C270R-mutated SARS-CoV-2 PL^{pro}-substrate complex. (b) The distribution of the PL^{pro}-substrate electrostatic and vdW interaction energy along the sidechain distance between R270 and P3-R collected from the frames of the MM/PBSA calculation of C270R-mutated SARS-CoV-2 PL^{pro}-substrate complex.

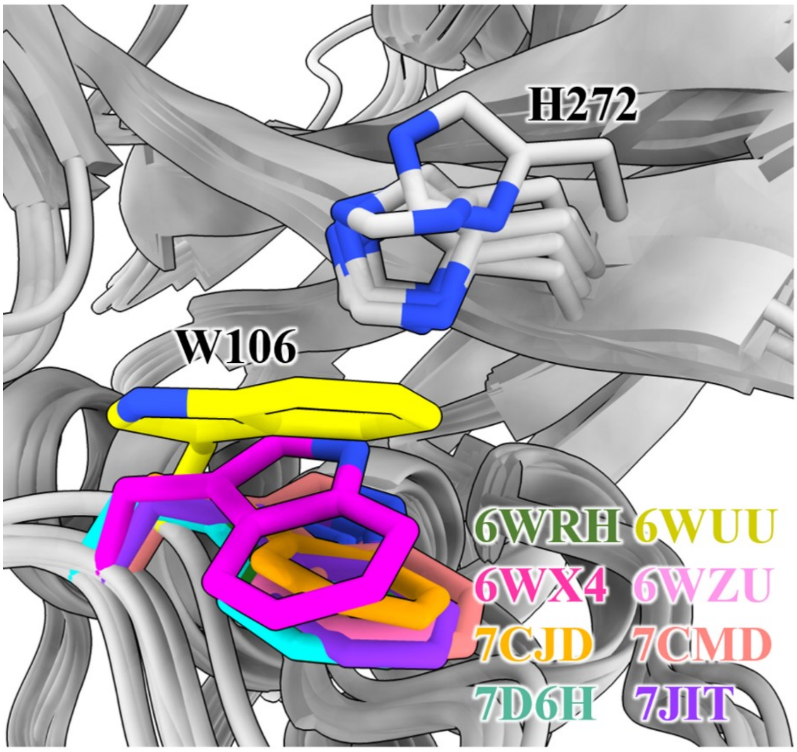

Fig. S16 Superposition of SARS-CoV-2 PL^{pro} crystal structures indicating the conformation diversity of W106 sidechain.

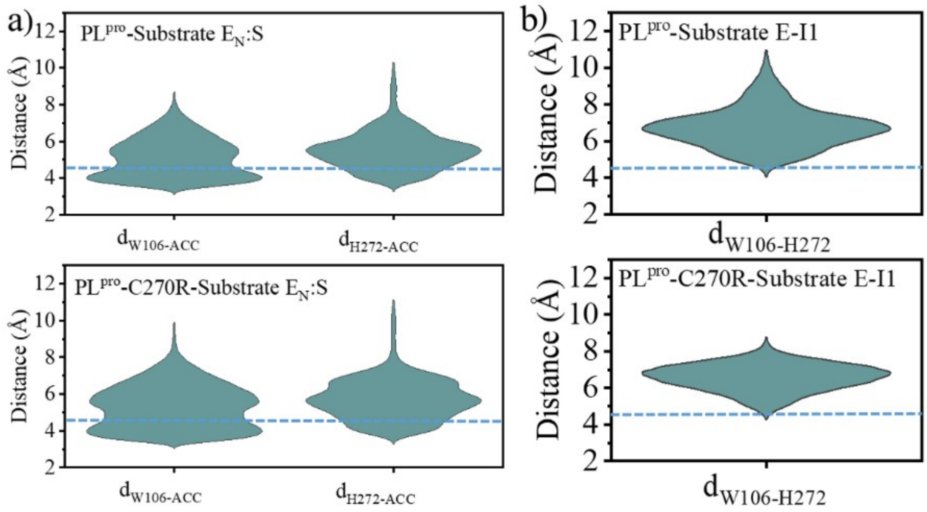

Fig. S17 (a) The ranges of the distance between the W106 sidechain and the coumarin skeleton of the ACC group of the substrate ($d_{W106\text{-}ACC}$), and the distance between the H272 sidechain and the coumarin skeleton of the ACC ($d_{H272\text{-}ACC}$) in the substrate-bound complexes of wild-type PL^{pro} and C270R mutant (E_N:S). (b) The ranges of the W106-H272 sidechain distances in the E-I1 intermediates of the wild-type PL^{pro} and C270R mutant. Dash lines at 4.5 Å indicate the formation of π-π interaction.

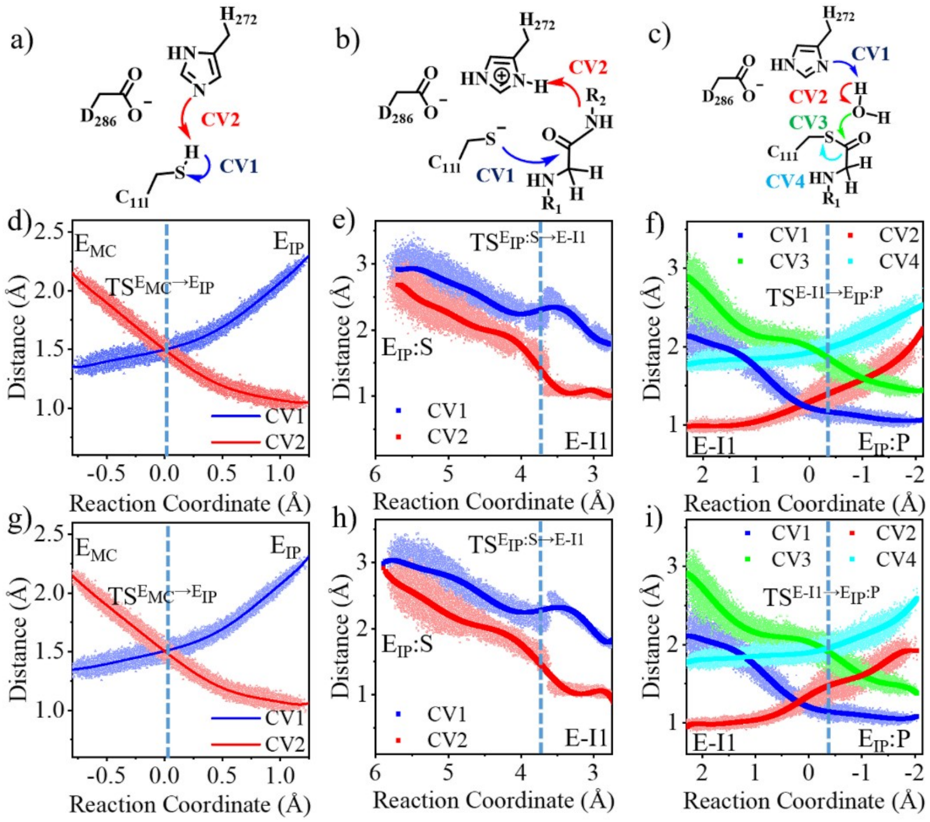

Fig. S18 (a-c) The schemes indicating the collective variables (CVs) for the reactions of C111–H272 proton transfer, acylation and deacylation. (d-f) Evolution of the CVs along the reactions for wild-type SARS-CoV-2 PL^{pro}. (g-i) Evolution of the CVs along the reactions for C270R mutated SARS-CoV-2 PL^{pro}. In each panel, a dash line represents the position of the transition state. The evolution of the CVs for the C111–H272 proton transfer is for apo PL^{pro}.

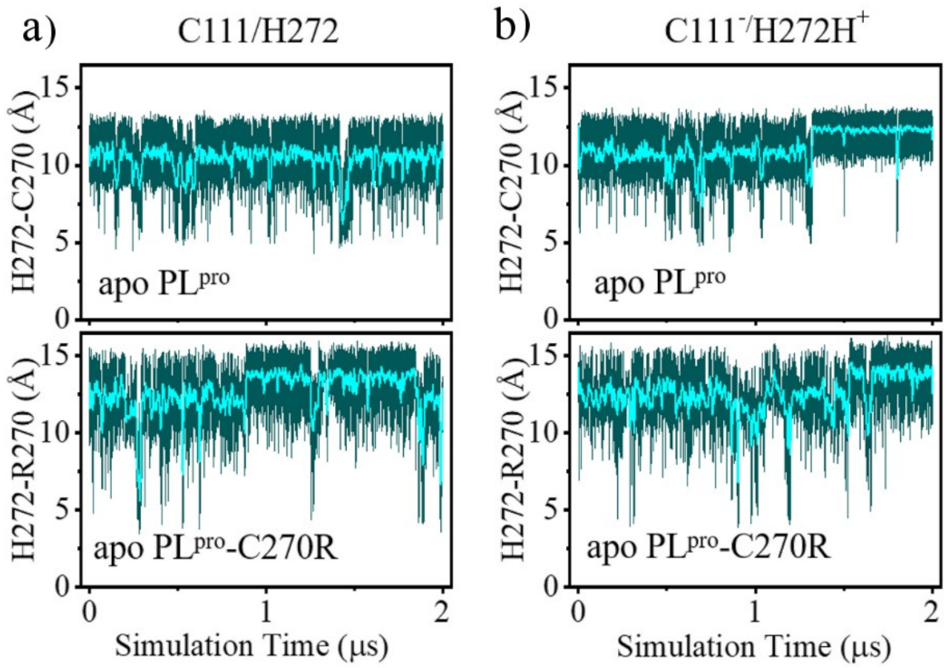

Fig. S19 GaMD simulation trajectories of apo wild-type SARS-CoV-2 PL^{pro} and C270R mutant containing (a) the neutral form of C111/H272 and (b) the ion pair form of $C111^{-}/H272H^{+}$. The trajectories are represented by the time series of the sidechain distance between H272 and C270/R270 (from top to bottom).

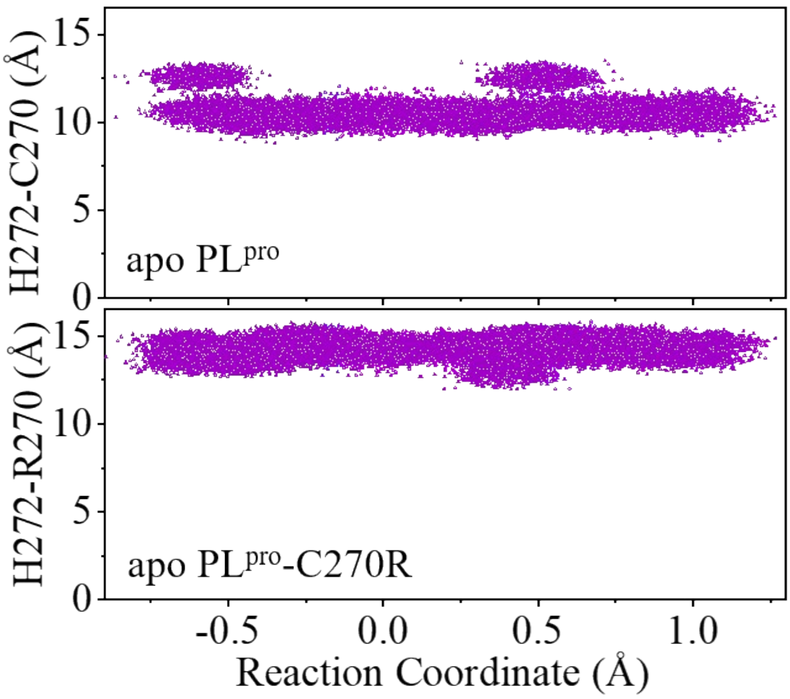

Fig. S20 The distribution of the sidechain distance of H272 to C270/R270 along the reaction coordinate of the C111–H272 proton transfer in the QM/MM systems of apo wild-type SARS-CoV-2 PL^{pro} and the C270R mutant.

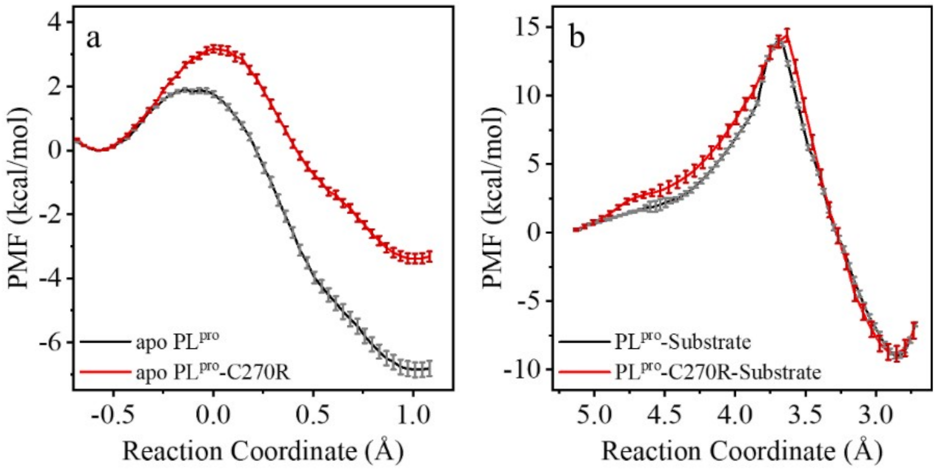

Fig. S21 Error bars estimating the QM/MM simulation convergence for (a) the C111–H272 proton transfer and (b) acylation reactions of the wild-type SARS-CoV-2 PL^{pro} and C270R mutant.

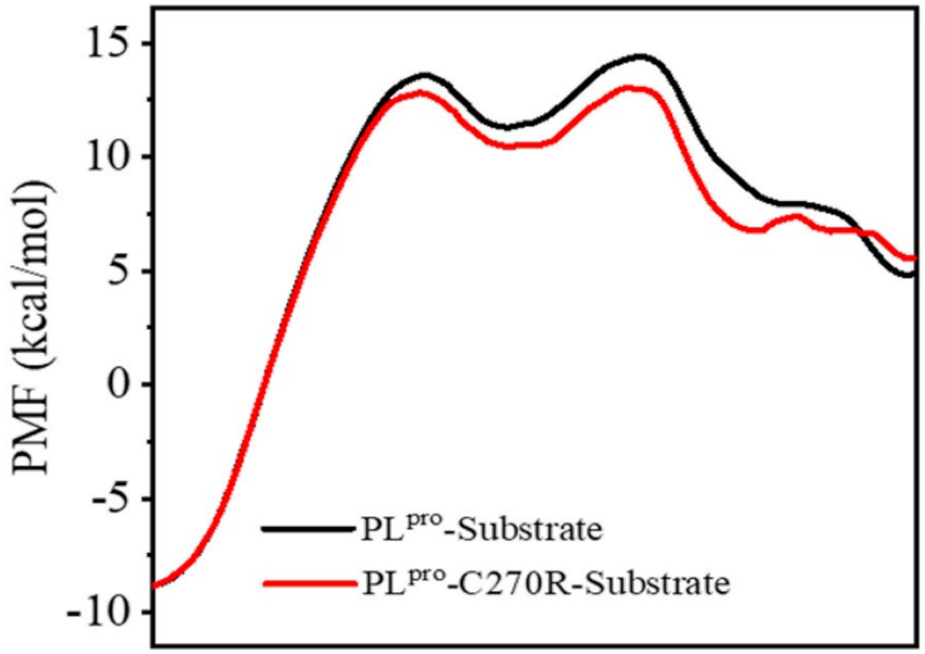

Fig. S22 Superposition of the QM/MM free energy profiles associated with the deacylation reaction as proposed in path I (Fig. 1) for the wild-type SARS-CoV-2 PL^{pro} and C270R mutant reacting with the substrate.
